# Aberrant RET expression affects normal mammary gland post-lactation transition, enhancing cancer potential

**DOI:** 10.1242/dmm.049286

**Published:** 2022-03-29

**Authors:** Sabrina A. Vallone, Martín García Solá, Carolina Schere-Levy, Roberto P. Meiss, Gladys N. Hermida, Lewis A. Chodosh, Edith C. Kordon, Nancy E. Hynes, Albana Gattelli

**Affiliations:** 1 Universidad de Buenos Aires (UBA), Facultad de Ciencias Exactas y Naturales, Ciudad Universitaria C1428EGA CABA, Buenos Aires, Argentina; 2 CONICET-UBA, Instituto de Fisiología, Biología Molecular y Neurociencias (IFIBYNE), Intendente Güiraldes 2160, Ciudad Universitaria, C1428EGA CABA Buenos Aires, Argentina; 3 Academia Nacional de Medicina de Buenos Aires, Departamento de Patología, Av. Gral. Las Heras 3092, C1425ASU CABA Buenos Aires, Argentina; 4 Departamento de Biodiversidad y Biología Experimental (DBBE), Biología de Anfibios-Histología Animal, Facultad de Ciencias Exactas y Naturales (FCEN), Buenos Aires, Argentina; 5 Department of Cancer Biology, Perelman School of Medicine, University of Pennsylvania, 614 BRB II/III, 421 Curie Blvd, Philadelphia, PA 19104, USA; 6 Friedrich Miescher Institute for Biomedical Research (FMI), Department of Emeritus, Maulbeerstrasse 66, CH-4058 Basel, Switzerland; 7 University of Basel, CH-4002 Basel, Switzerland

**Keywords:** Breast cancer, Involution, Mammary gland, RET, Stat3

## Abstract

RET is a receptor tyrosine kinase with oncogenic potential in the mammary epithelium. Several receptors with oncogenic activity in the breast are known to participate in specific developmental stages. We found that RET is differentially expressed during mouse mammary gland development: RET is present in lactation and its expression dramatically decreases in involution, the period during which the lactating gland returns to a quiescent state after weaning. Based on epidemiological and pre-clinical findings, involution has been described as tumor promoting. Using the Ret/MTB doxycycline-inducible mouse transgenic system, we show that sustained expression of RET in the mammary epithelium during the post-lactation transition to involution is accompanied by alterations in tissue remodeling and an enhancement of cancer potential. Following constitutive *Ret* expression, we observed a significant increase in neoplastic lesions in the post-involuting versus the virgin mammary gland. Furthermore, we show that abnormal RET overexpression during lactation promotes factors that prime involution, including premature activation of Stat3 signaling and, using RNA sequencing, an acute-phase inflammatory signature. Our results demonstrate that RET overexpression negatively affects the normal post-lactation transition.

## Abstract (precis)

**Summary:** We show that RET activation stimulates Stat3 signaling in mammary epithelial cell culture and *in vivo* during post-lactation transition, demonstrating that the RET receptor participates in the post-lactation transition priming tumorigenesis.

## INTRODUCTION

Cancer can be viewed as a disease of defective development, wherein the signaling processes that guide normal tissue growth and morphogenesis become deregulated to facilitate cancer cell proliferation. To understand the mammary gland tumorigenic process, normal biology of the organ is probed. Members of the receptor tyrosine kinase (RTK) family are known breast oncoproteins, e.g. ERBB2 and FGFR, and are known to have specific functions during development (Hynes and Watson, 2010). Using a doxycycline (DOX)-inducible transgenic mouse system (Ret/MTB) [where MTB indictes MMTV-rtTA transgenic mice (Gunther et al., 2002)] we have previously demonstrated that the RTK RET is oncogenic in the mammary epithelium (Gattelli et al., 2018). We identified *RET* gene overexpression as a breast cancer driver; however, its function in the mammary gland remains understudied. Here, we show that RET protein is expressed during lactation and abnormal RET expression negatively impacts the post-lactation transition, inducing early markers of cancer.

The mammary gland undergoes cycles of cell proliferation in puberty and pregnancy, terminal differentiation in lactation and regression in involution (Watson and Khaled, 2008), when tissue remodeling occurs and the mammary gland transitions to a quiescent state similar to the virgin gland (Stein et al., 2007). Involution is divided into two distinct phases. The first phase (0-48 h after weaning in mice) is initiated by local factors triggered by milk accumulation in the gland due to cessation of suckling (Li et al., 1997; Quaglino et al., 2009). In this situation, cell and tissue architecture are maintained, involution is reversible, and lactation resumes with suckling. The second phase (from 48 h to ∼120 h) is regulated by systemic hormones and is irreversible; tissue architecture is disturbed and a robust remodeling is accompanied by removal of milk and dead cells as well as adipogenesis (Akhtar et al., 2016)*.* At the end of this phase, the mammary gland is filled with adipocyte tissue (Baxter et al., 2007)*.* Apoptosis, triggered by detachment of luminal epithelial cells from the basement membrane, occurs during involution (Debnath, 2008). Mammary gland involution is an abrupt tissue-remodeling process also associated with an inflammatory acute-phase response (Pennock et al., 2018; Stein et al., 2004). In the clinic, this process is of particular interest because post-lactation transition to involution has been proposed to contribute to breast cancer progression (Lyons et al., 2011; McDaniel et al., 2006; Pennock et al., 2018; Schedin et al., 2007; Watson, 2006). Interestingly, in ∼50% of young women, there is an association with post-partum breast cancer (Callihan et al., 2013; Lima et al., 2020), which has poor prognosis.

Regulation of cell proliferation and apoptosis in the mammary gland proceeds mainly through the action of the signal transducers and activators of transcription (Stat) family of proteins (Haricharan and Li, 2014; Watson, 2001). Stats are activated by phosphorylation (p-Stat), mainly at tyrosine residues (Bromberg and Darnell, 2000). Stat1, Stat3 and Stat5 have been well studied during mammary gland development (Haricharan and Li, 2014). Stat1 is phosphorylated in virgin and in involuting glands (starting at 72 h after weaning) (Haricharan and Li, 2014; Watson, 2001). During early pregnancy, Stat5 is activated to regulate mammary gland proliferation and later lactation (Clarkson et al., 2004). At weaning, Stat5 is rapidly inactivated (Liu et al., 1997), and a Stat3-mediated apoptotic program is initiated to clear the mammary gland of excess cells. During the first phase of involution, p-Stat3 levels are rapidly elevated, which is essential for involution (Chapman et al., 1999; Humphreys et al., 2002). During the second phase, active Stat3 is responsible for inducing an immune response, polarizing inflammatory cells required for epithelial cell clearing (Hughes et al., 2012; Kreuzaler et al., 2011). Targets of Stat3 signaling include pathways of lysosome-mediated cell death involving cathepsins (Kreuzaler et al., 2011) and mitochondrial-mediated caspase activation inducing apoptosis (Li et al., 1997). Several reports indicate that the levels of Stat1, Stat3 and Stat5 activation affect mammary tumor development (Pensa et al., 2009; Rädler et al., 2017). It was reported that molecular changes associated with Stat3-induced apoptosis include downregulation of PI3K/Akt pro-survival signaling activity (Abell et al., 2005). Other reports showed that Akt expression leads to the sustained survival of mammary epithelial cells during involution, despite strong Stat3 activation (Creamer et al., 2010; Li et al., 2002). In contrast to tight regulation in mammary epithelial cells, Stat3 signaling in breast cancer is dysregulated (Hughes and Watson, 2018), and, although indirect, it is active downstream of ERBB2 and RET (Gattelli et al., 2013; Hynes and Watson, 2010).

The glial-derived neurotrophic factor (GDNF) family of peptides (GFLs) are RET ligands that bind RET together with GDNF receptor α family co-receptors (GFRα) (Ibáñez, 2013). GFRα homodimers are recruited by specific GFLs into a high-affinity complex, which interacts with and activates RET homodimers leading to subsequent autophosphorylation (Ibáñez, 2013). RET activation promotes cancer progression in several tumor types (De Groot et al., 2006; Drilon et al., 2018; Mulligan, 2019). Although *RET* genomic alterations have been described in breast cancer (Paratala et al., 2018), results from independent studies, including our own, demonstrate that *RET* gene overexpression plays a key role in breast cancer pathogenesis and therapy response (Gattelli et al., 2020; Spanheimer et al., 2014; Varešlija et al., 2019). We have previously shown that high RET levels in primary breast tumor samples correlate with decreased overall survival (Gattelli et al., 2013). Using different RET-dependent breast cancer models, including the Ret/MTB transgenic mouse (Gattelli et al., 2018), we have reported that pharmacological inhibition of RET activity or *Ret* downregulation, reduces cell proliferation, inflammation and metastatic potential (Boulay et al., 2008; Gattelli et al., 2013, 2018), and that RET-driven tumor outgrowth is accompanied by increased levels of Stat3 and also Stat1 phosphorylation (Gattelli et al., 2013, 2018). In this study, we show that RET activation stimulates Stat3 signaling in mammary epithelial cell culture and *in vivo* during post-lactation transition. Collectively, our data demonstrate that abnormal RET expression drives the post-lactation process towards tumorigenesis.

## RESULTS

### RET expression is regulated during mammary gland development

**Fig. 1.**
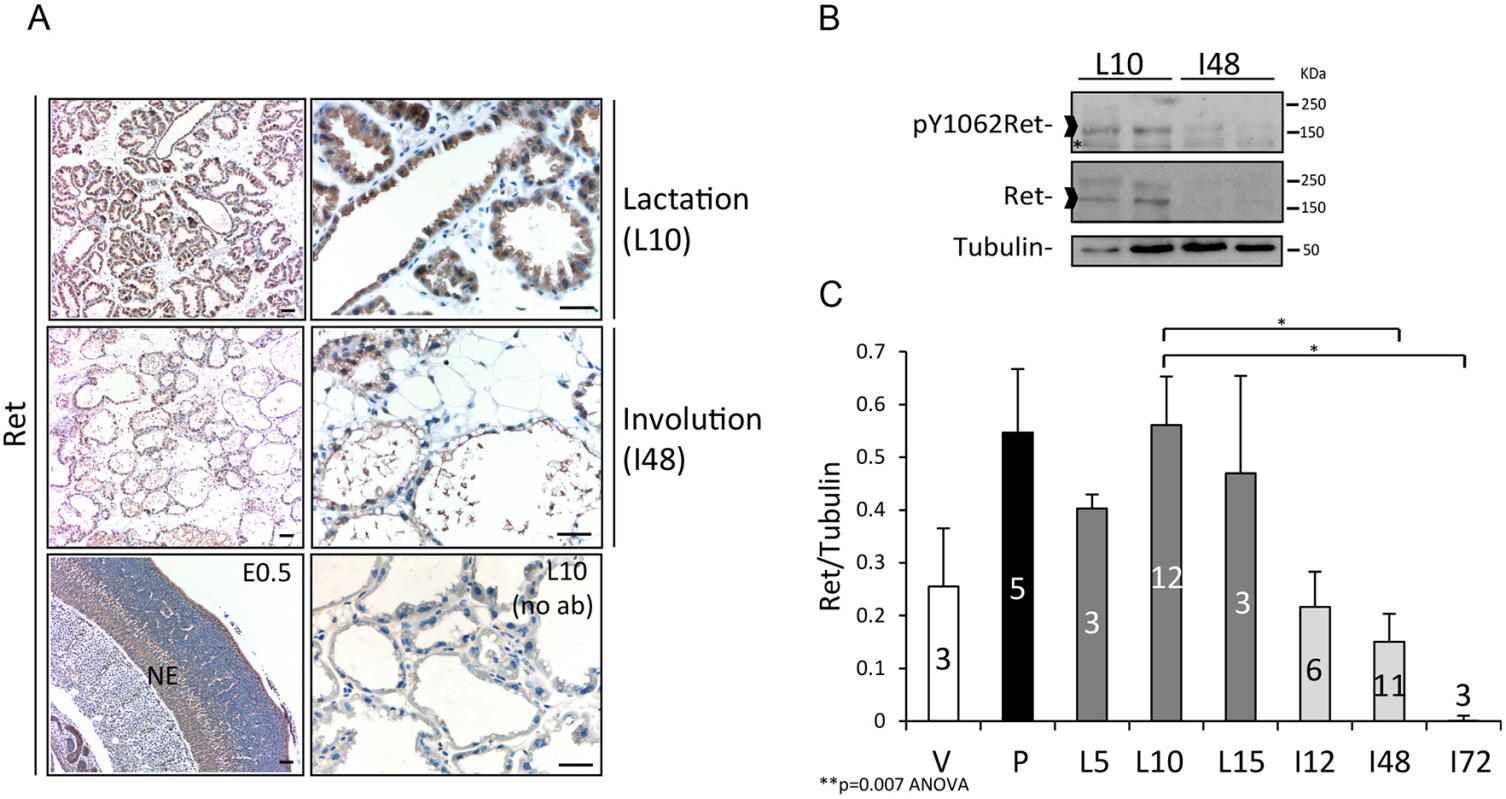
**RET receptor expression during mammary gland development.** (A) Sections of mammary gland tissue from FVB/N females at developmental stages lactation day 10 (L10) or 48 h of weaning-induced involution (I48) were stained by immunohistochemistry (IHC) using a RET-specific antibody. Representative pictures are shown. A section from a mouse embryo [embryonic day (E)0.5] was used as a positive control for RET staining in the neuroepithelium (NE); a 10 day lactating gland (L10) without primary antibody (no ab) was used as a negative control. Scale bars: 50 µm. (B) Western blot (WB) analysis of total RET protein and its tyrosine 1062 phosphorylated form (p-Y1062RET). Mammary lysates from FVB/N wild-type adult females at the indicated developmental stages were analyzed. Tubulin protein was used as loading control. An unspecific band in the p-Y1062RET samples is indicated by an asterisk. Carets indicate the specific band used for RET quantification. (C) RET protein levels relative to tubulin were determined by WB quantification using ImageJ on mammary lysates for the indicated developmental stages. The results are shown as the mean±s.e.m. *P*-values were calculated by one-way ANOVA (**P=0.007) and Tukey's multiple comparisons test (*P<0.05). The number of animals used for each developmental stage is indicated on bars (*n*=3-12). Designation of stages: V, virgin: 2-month-old adult virgin female; P, pregnancy: at day 15 or day 18 post-conception; L, lactation: L5 to L15, lactation at day 5, day 10 or day 15 of the first lactation period; I, involution: I12 to I72, involution period 12, 48 or 72 h after pup withdrawal.

The RET receptor has important functions in multiple cell types (De Groot et al., 2006; Perea et al., 2017); however, its potential role in the mammary gland has not been explored and we addressed this as follows. Mammary tissues at different developmental stages were analyzed by immunohistochemistry (IHC) and western blotting (WB) using RET-specific antibodies (Fig. 1). IHC revealed that RET protein is present in 10 day-lactating mammary epithelium (L10), a time of established lactation. In contrast, no RET protein was evident in luminal cells from post-lactating glands 48 h after onset of weaning-induced involution (I48) (Fig. 1A). These findings are consistent with published data showing that the *Ret* gene is most highly expressed in lactating mammary glands (Bambhroliya et al., 2018; Clarkson et al., 2004; Grinman et al., 2019; Stein et al., 2004). Analysis of mammary tissue lysates by WB revealed that glands from lactating animals exhibit high levels of active RET protein (phosphorylated RET) compared to glands from post-lactating animals (Fig. 1B). We also found expression of the RET ligands and co-receptors during lactation and involution (Fig. S1A), suggesting that the RET network is functional.

The post-lactational period represents a developmental window contributing to breast cancer risk (Fornetti et al., 2014). To examine this phase more closely, we performed a kinetic analysis of RET protein expression focusing on lactation and the two phases of involution (Fig. 1C). RET expression peaks at pregnancy (P) and remains high from day 5 through 15 days of lactation (L5, L10 and L15). By 12 h of involution (I12), RET expression levels rapidly decrease and remain significantly lower 48 h and 72 h (I48 and I72, respectively) after weaning (Fig. 1C; Fig. S1B). Thus, RET expression begins during pregnancy, remains high during lactation and rapidly decreases within 12 h of involution, suggesting its tight control at the post-lactation transition.

**Fig. 2.**
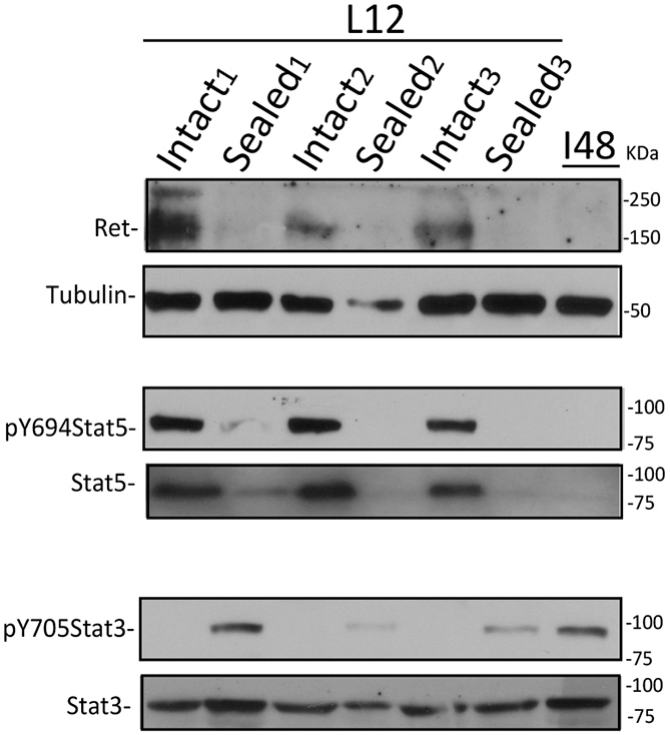
**RET expression is regulated locally by milk stasis.** RET expression levels as determined by WB using #4 mammary gland lysates from three independent nursing females (labeled 1-3) in which, at 10 days of lactation, the right glands were left intact (Intact, *n*=3) or milk efflux from the contralateral glands was blocked by sealing the nipple (Sealed, *n*=3). The females continued nursing their pups for an additional 48 h (L12). As a control, RET expression levels in lysates from a 48 h weaning-induced involuting gland of a control female were assayed by WB (I48). WB analysis for Stat3 and Stat5 activation using the indicated antibodies is shown. Tubulin detection was used as a loading control.

RET regulation at post-lactation was further explored using wild-type (WT) FVB/N female mice with a unilaterally sealed inguinal mammary gland teat to remove the suckling stimulus (Li et al., 1997; Schere-Levy et al., 2003). For this, one of the #4 mammary glands of 10 day-lactating females was sealed, while the other nine glands remained intact. Using this technique, lactogenic hormones remain at their systemic level, while involution-associated local factors are triggered in the sealed gland. The experimental mice continued nursing their pups for an additional 48 h (L12) then both #4 glands were removed. Our results clearly show that sealing leads to downregulation of RET expression (Fig. 2). As readout for normal mammary development, we examined Stat activation. Intact glands show high levels of p-Stat5 and no p-Stat3, while lysates from an involuting mammary gland (I48) show the reverse, high levels of p-Stat3 and no p-Stat5 (Fig. 2). Milk stasis is known to cause Stat3 activation (Li et al., 1997; Schere-Levy et al., 2003), which we observed in the sealed glands. Thus, our results show a correlation between RET expression and the transition to involution, and, importantly, the initial reduction of RET at involution onset is not due to systemic decreases in circulating lactogenic hormones, e.g. prolactin and hydrocortisone (Nguyen and Pollard, 2000), but responds to local signals induced by lactation cessation.

### Abnormal RET expression alters the involution process

**Fig. 3.**
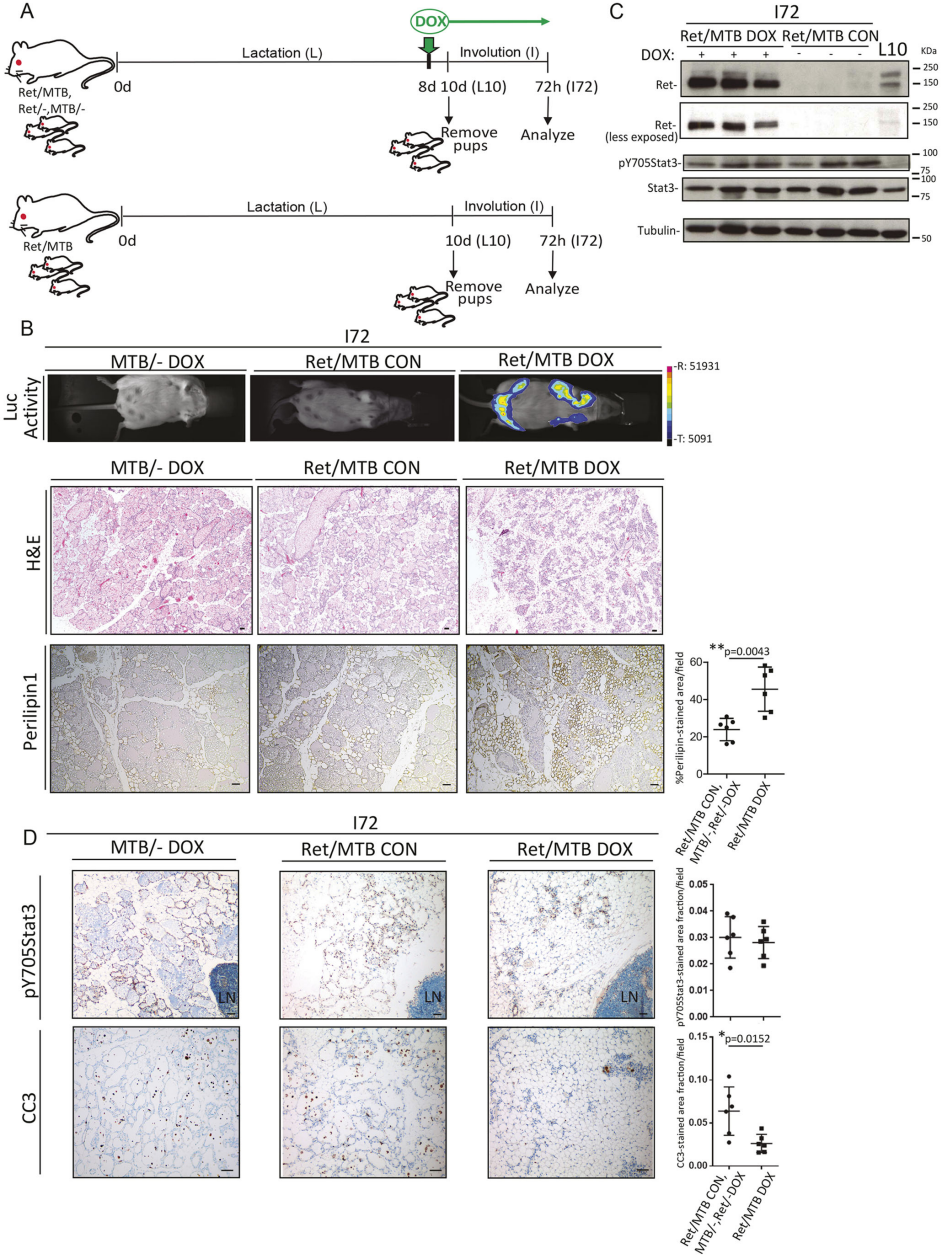
**RET expression in the post-lactation transition causes precocious involution.** (A) Schematic overview of the timing of doxycycline (DOX)-driven *Ret* induction (top) or control (bottom) in Ret/MTB, Ret/−, MTB/− strains (top) or non-DOX treated Ret/MTB mice. (B) Mammary tissue of glands from the indicated mice at 72 h involution (I72) were monitored for luciferase activity (Luc Activity) to check *Ret* transgene expression in the experimental compared to control animals. R, range; T, threshold. On the indicated sections, H&E staining and IHC for specific perilipin-1 staining was performed. Data are presented as the mean±s.e.m. (*n*=6 in each group). Quantification corresponding to two independent experiments of the indicated stain is shown. *P*-values by Mann–Whitney test. (C) WB shows RET and p-Y705Stat3 levels using lysates from 72 h-involuting mammary gland tissue taken from the indicated strains. Mammary tissue lysate from a 10 day-lactating female (L10) was included to control for normal endogenous RET expression levels. Phospho- and total Stat3 levels were determined on these samples. Tubulin was used as a loading control. (D) IHC analysis was performed on sections for specific p-Y705Stat3 or CC3 staining. Data are presented as the mean±s.e.m. (*n*=6 in each group), and corresponding quantifications for two independent experiments of the indicated stain are shown. *P*-values by Mann–Whitney test. LN, inguinal lymph node. Scale bars: 50 μm.

Using the Ret/MTB mouse model, we have previously shown that chronic overexpression of WT *RET* gene in the mammary epithelium induces hyperplasia and mammary tumors (Gattelli et al., 2018). In this system, Ret expression is DOX inducible, allowing its conditional expression during discrete stages of mammary development. In the next experiments, we conditionally expressed Ret during the early involution phase, when its levels drop after lactation. A scheme of the experiment is shown in Fig. 3A. After 8 days’ lactation, Ret was induced in bitransgenic Ret/MTB parous females by administration of DOX, and pups were removed 2 days later, which is considered involution start. Single transgenic MTB/− or Ret/− females under DOX treatment (DOX) (Fig. 3A, top) or non-induced Ret/MTB females under normal feeding (CON) (Fig. 3A, bottom) were used as control groups. *Ret* transgene expression was monitored *in vivo* using bioluminescence in all animal groups (Fig. 3B, top row). After 72 h of involution (I72), mammary glands were harvested and analyzed. WB confirmed that lysates from DOX-treated (DOX), but not control (CON) mice showed high levels of RET (Fig. 3C). Hematoxylin and Eosin (H&E) staining of sections from I72 glands revealed a significant increase in the adipose tissue area in the RET-expressing glands compared to controls (Fig. 3B, middle row; Fig. S2A), indicating a more advanced involution process. Quantification of adipocyte-specific perilipin-1 levels (Hume et al., 2018), which is significantly increased in RET-expressing glands (Fig. 3B, bottom row), verified the increase in adipose area. These glands were also examined for other markers of involution: cleaved caspase 3 (CC3; also known as CASP3) and p-Stat3 (Fig. 3D). Despite the histological differences observed, the p-Stat3 levels were similar in I72 glands from controls and RET-expressing female mice. Interestingly, staining for CC3 revealed a significant reduction in the number of dead cells in the RET-expressing glands (Fig. 3D), as it is observed in a more advanced involution stage (Fig. S2B). These results indicate that the tissue remodeling changes induced by continuous RET expression during early phases of the post-lactation transition alter the normal kinetics of involution, as seen for the decrease in CC3 and the increase in adipocyte filling, thereby causing an accelerated involution phenotype.

### Sustained post-lactation RET expression promotes developmental defects, increasing malignancy

**Fig. 4.**
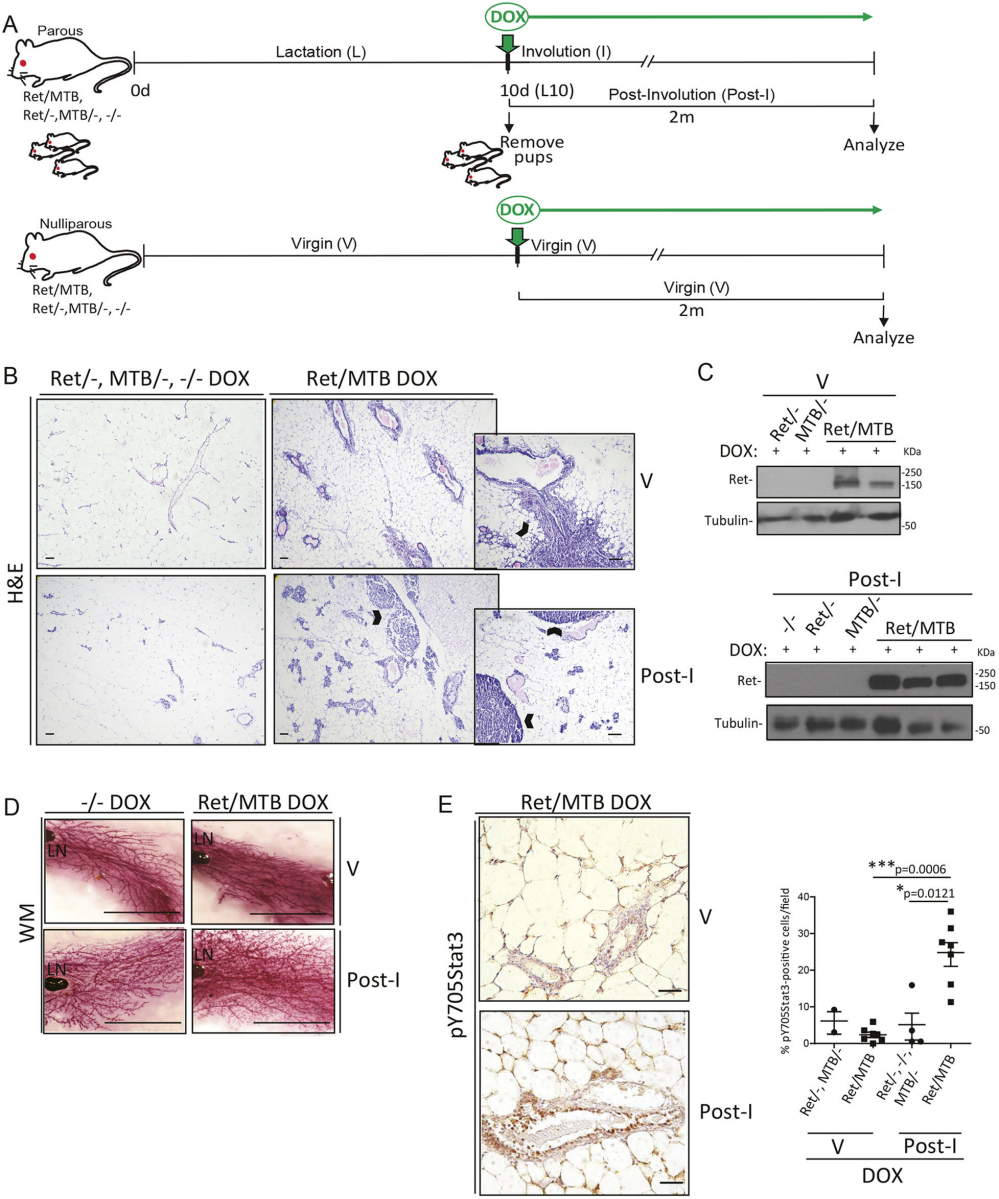
**Continuous RET expression during post-lactation transition enhances cancer potential.** (A) Schematic representation of chronic 2 month DOX-driven *Ret* induction in the Ret/MTB mouse model. Single parous (top) and nulliparous (bottom) virgin (V) adult mice of the indicated genotypes were used. DOX induction started as indicated by green arrows for each control (Ret/−, MTB/− or −/−) and experimental groups (Ret/MTB). The animals were kept under DOX (2 g/l) treatment for 2 months (2m). Mammary glands from control and experimental groups in conditions of both nulliparous (V) or parous after finishing the involution period (Post-I) were analyzed. (B) H&E-stained sections of the indicated mice were analyzed and more mammary intra-epithelial neoplasia (MIN), indicated by carets, were found in the RET-overexpressing glands from post-lactation compared to virgin glands. Scale bars: 50 μm. (C) WB shows RET levels for lysates of mammary gland tissue from representative females of these experiments. (D) Mammary glands at the indicated conditions were analyzed by whole-mount preparations. Representative pictures for each condition showing the lymph node (LN) are shown. Scale bars: 1.25 cm. (E) IHC for p-Y705Stat3 was performed on mammary gland sections from virgin (V: Ret/− or MTB/− *n*=2, Ret/MTB *n*=7) or post-involuting (Post-I: Ret/−, MTB/− or −/− *n*=4, Ret/MTB *n*=7) glands. Data are presented as the mean±s.e.m. Quantification corresponding to three independent experiments is shown. *P*-values by Mann–Whitney test. Scale bars: 50 μm.

To further analyze the impact of sustained RET expression during the transition from lactation to involution, a period in which the mammary gland environment experiences distinct features promoting tumorigenesis (Lyons et al., 2011; McDaniel et al., 2006; Pennock et al., 2018), we extended the time of its DOX-induced expression up to 2 months (Fig. 4A), the time at which hyperplasia develops (Gattelli et al., 2018). H&E staining revealed a significant increase in the number of mammary intra-epithelial neoplasia (MIN) present in the 2 month DOX-induced RET-expressing Ret/MTB glands that experience the post-involution process (Ret/MTB Post-I), in comparison to RET-expressing Ret/MTB virgin glands (Ret/MTB V) (Table 1, Fig. 4B). *Ret* transgene expression was confirmed in mammary tissue by WB (Fig. 4C).

**Table 1.**
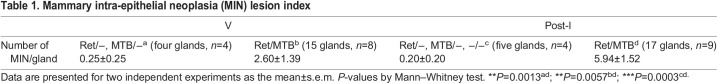
Mammary intra-epithelial neoplasia (MIN) lesion index

Inspection of H&E-stained histological sections showed that, in comparison to the Ret/MTB hyperplastic mammary glands from nulliparous (Ret/MTB V) females, the Ret/MTB mammary glands from Post-I females (Ret/MTB Post-I) exhibit pronounced aberrant morphology, consisting of ductal enlargement containing milk protein β-casein, so-called ectatic ducts, surrounded by persistent alveoli, as well as aberrant stroma with areas of brown-like fat (Fig. S2C,D). Control Post-I glands (Ret/− or MTB/− Post-I) show normal post-involuting morphology with some residual alveoli (Fig. 4B). Morphology is also shown in the whole mounts (Fig. 4D). Taken together, these results provide evidence that aberrant RET expression affects the tissue-remodeling process, promoting neoplastic lesion formation during post-lactation.

Next, we analyzed Stat3 activation in the experimental mice. The results show that RET-expressing post-involuting glands (Ret/MTB Post-I) have a significant increase in active Stat3 levels, compared to control virgin glands (Ret/MTB V) (Fig. 4E). We and others have reported that Stat3 is activated in Ret-driven mammary tumors (Gattelli et al., 2013, 2018; Plaza-Menacho et al., 2011). These new data demonstrate that, within 2 months of RET overexpression in the Ret/MTB glands, increased Stat3 activity is evident, which likely plays a role favoring tumorigenesis. We confirmed *in vitro* that RET activation stimulates Stat3 activity in mammary epithelial cells. Following RET stimulation with GDNF, non-transformed HC11 mammary epithelial cultures, which express *Ret* and its co-receptors, showed an increase in p-Stat3 levels (no p-Stat5) as well as in RET canonical proliferation markers, p-Erk (also known as p-MAPK) and p-Akt (Fig. S3).

### RET overexpression during lactation induces premature involution markers and regulates early involution-associated genes that increase tumor risk

**Fig. 5.**
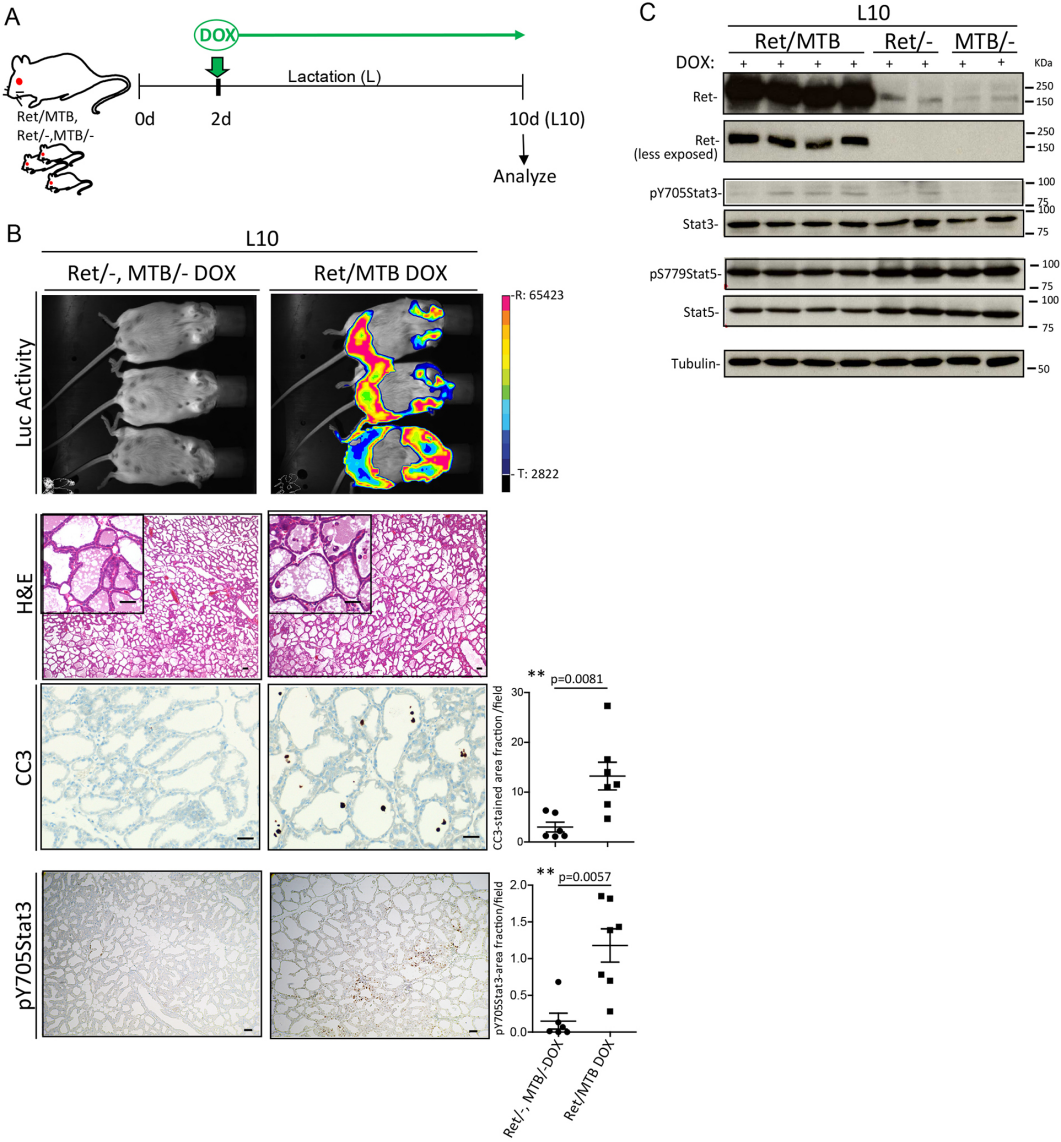
**RET overexpression in lactation promotes involution-associated markers.** (A) Schematic overview of the DOX-driven *Ret* induction in the engineered Ret/MTB mouse model. (B) Mammary gland sections from Ret/− or MTB/− (*n*=6) and Ret/MTB (*n*=7) females at 10 days of lactation (L10) following treatment with DOX (2 g/l) for 8 days were analyzed by H&E and IHC for CC3 and p-Y705Stat3. Data are presented as the mean±s.e.m. Scale bars: 50 μm. Quantifications corresponding to two independent experiments of the indicated gland stain are shown. *P*-values by Mann–Whitney test. Mice were monitored for luciferase activity (Luc Activity) as a control for *Ret* transgene expression. R, range; T, threshold. (C) WB shows RET levels at two different exposure times and p-Stat signaling for lysates from mammary gland tissue from the experiments showed in B. Tubulin was used as a loading control.

Despite the fact that an increased risk of developing breast cancer is observed in post-partum women, a protective effect of lactation against breast cancer has been reported for young (<25 years) child-bearing females (Borges et al., 2020; Schedin, 2006). To investigate the consequence of triggering RET expression during this protective period, we induced *Ret* gene overexpression during lactation by providing DOX-containing drinking water to Ret/MTB lactating females between day 2 and 10, the day on which glands were harvested (Fig. 5A). RET expression was monitored using bioluminescence (Fig. 5B). H&E staining revealed the presence of detached cells only in the alveoli of lactating mammary glands from Ret/MTB females (Fig. 5B, insets), suggesting that they undergo premature cell death. Supporting this, IHC showed a significant increase in CC3-positive cells in 10 day-Ret/MTB-induced glands but not in control glands (Fig. 5B, right column of images). We also detected a significant increase in p-Stat3 levels (Fig. 5B, bottom row). RET overexpression in the DOX-induced Ret/MTB bitransgenic glands was confirmed by WB (Fig. 5C), wherein a significant induction of p-Stat3 (not p-Stat5) was observed (Fig. S2E). These results strengthen the conclusion that aberrant RET overexpression induces premature involution.

**Fig. 6.**
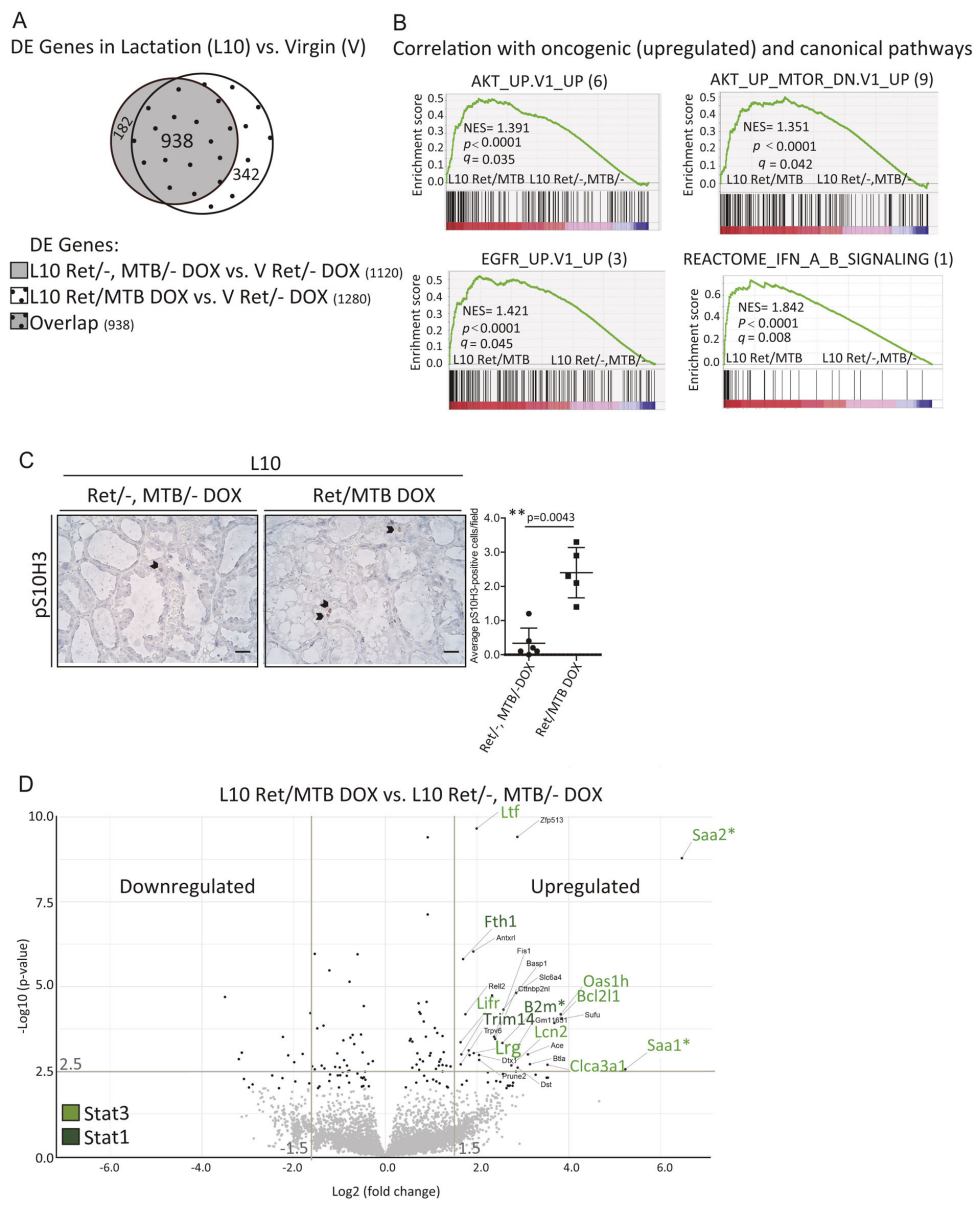
**RET promotes acute inflammatory response involution-associated gene expression.** (A) RNA-sequencing data analysis of mammary glands from lactation day 10 (L10) DOX-induced Ret/MTB females (L10 ret/MTB DOX) or control Ret/− and MTB/− females (L10 Ret/−, MTB/− DOX) in comparison to virgin (V) DOX-treated control Ret/− animals (V Ret/− DOX) is presented as a Venn diagram of the number of differentially expressed (DE) genes. *P*≤0.01; *P*-values were calculated as implemented in R (*n*=6 L10 Ret/MTB mice, *n*=3 L10 Ret/− and *n*=3 L10 MTB/− mice, *n*=4 V Ret/– mice). (B) Gene set enrichment analysis (GSEA) was carried out using publicly available sets (MSigDB). GSEA shows the correlation with the gene sets upregulated with more than 120 genes in size between the top ten oncogenic pathways: top three (3) EGFR_UP.V1_UP, top six (6) AKT_UP.V1_UP and top nine (9) AKT_UP_MTOR_DN.V1_UP, in the six Ret/MTB in contrast to the six control 10 day-lactating glands (Ret/−, MTB/−). The bottom-right GSEA shows the correlation between the top (1) canonical pathway, REACTOME_INTERFERON_ALPHA_BETA_SIGNALING, in the same conditions. Peaks or valleys that occur on the edges represent pathways differentially regulated by RET overexpression. NES, normalized enrichment score; *q*, false-discovery rate. *P*-value was computed as implemented in GSEA. (C) IHC for p-S10H3 staining and the corresponding quantification was performed on sections of mammary glands of Ret/MTB or control (Ret/−, MTB/−) DOX-induced females on lactation day 10 (L10). Data are presented as the mean±s.e.m. Scale bars: 50 μm. (D) Volcano plot showing gene expression of RET-expressing bitransgenic mammary glands (Ret/MTB, *n*=6) compared with control mammary glands (Ret/−, *n*=3 and MTB/−, *n*=3) from 10 day-lactating females on DOX treatment (L10 Ret/MTB DOX versus L10 Ret/−, MTB/− DOX). Black dots correspond to the 149 significantly deregulated genes (*P*≤0.01); at the bottom, the non-significant genes are shown as gray dots. Gene symbols of the most significantly upregulated genes are indicated [Log2(fold change)≥1.5; –Log10(*P*)≥2.5; *P*≤0.01]. From the literature, we highlighted genes by color code that are Stat3 (or Stat1) regulated in mammary epithelium. Genes described as post-partum-associated high breast cancer risk are indicated by asterisks.

We noted that p-Stat3 staining was clustered in certain areas of the RET-overexpressing lactating glands (Fig. 5B, bottom row), suggesting that Stat3 activation might be an indirect consequence of RET-triggered local factors, which could also impact the microenvironment. To explore the underlying mechanism, we performed RNA sequencing on mammary gland tissue from 10 day-lactating (L10) RET-overexpressing Ret/MTB females (*n*=6) and 10 day-lactating (L10) Ret/− and MTB/− control females (for both, *n*=3). All animals were under DOX induction. Mammary tissue of DOX-treated virgin (V) Ret/− females (*n*=4) was also analyzed. Comparing gene expression in L10 Ret/−, MTB/− mice to that in V Ret/− females demonstrated that 1120 (182+938) genes were differentially expressed (Fig. 6A); 1280 (938+342) genes were differentially expressed in L10 RET-overexpressing Ret/MTB glands compared to V control glands (938 genes overlapped) (Fig. 6A). Finally, 182 genes and 342 genes were specifically expressed in L10 Ret/−, MTB/− control females and in L10 Ret-overexpressing Ret/MTB females, respectively. These data demonstrate that lactation is associated with major changes in gene expression (73.28%, 938/1280) and that RET upregulation at this stage causes dysregulation of a smaller fraction of genes (26.72%, 342/1280) (Fig. 6A). We observed no differences in the ability of RET-overexpressing females to nurse their pups in comparison to control females, suggesting that this developmental process proceeds normally.

Next, we used gene set enrichment analysis (GSEA) to characterize RET-associated gene expression in L10 RET-overexpressing Ret/MTB mammary glands compared to L10 control glands. Pathway enrichment using the MSigDB hallmark gene set collection (Liberzon et al., 2015) showed significant differences in upregulated oncogenic pathways that were enriched in Akt (Fig. 6B), a survival signal driven by RET (Gattelli et al., 2013). There was also a significant increase in the proliferation marker phosphorylated histone 3 (p-H3) in RET-overexpressing lactating glands (Fig. 6C), showing that RET is able to deregulate cell fate at this stage. In addition, performing GSEA on canonical pathways showed that the top signature corresponded to interferon (IFN) response (Fig. 6B) and a significant enrichment in biological process and transcription factors related to the IFN type I response (Fig. S4A). The IFN pathway is associated with acute inflammation, and key players in this pathway are the Stat proteins (Haricharan and Li, 2014). This result also strengthens the conclusion that RET overexpression in lactation causes a premature involution phenotype. Moreover, these results may be mechanistically relevant because gene expression in the first days of involution resembles an acute-phase inflammatory response (Clarkson et al., 2006; Hughes et al., 2012; Stein et al., 2004), which, at later times, is replaced by a wound-healing-like immune signature (Martinson et al., 2015).

To examine this further, we compared transcripts in L10 RET-overexpressing Ret/MTB glands to L10 control mammary glands, which revealed differential expression of 149 genes (Table S1). A list of the top genes up- and downregulated (2LogFC≥1.5; *P*≤0.01) is shown in Table 2. Several genes corresponding to the first phase of involution and Stat3 activity are upregulated (Clarkson et al., 2006; Hughes and Watson, 2018). These include serum amyloid A1 and A2 genes (*Saa1*, *Saa2*), genes encoding members of the 2′-5′ oligoadenylate synthetase enzyme family (e.g. *Oas1h*) and genes encoding calcium-related proteins (e.g. *Clca3a1*). Other genes related to survival and inflammation including the anti-apoptotic Bcl2-like 1 (*Bcl2l1*), leucine-rich 2-glycoprotein 1 (*Lrg1*) and Lif receptor (*Lifr*) were also identified (Table 2). Stat1 is also activated in involution (Haricharan and Li, 2014), and, in addition to known Stat3-target genes in mammary epithelium, we identified Stat1-induced genes, e.g. β2 microglobulin (*B2m*) (Radisky and Hartmann, 2009). These are highlighted in the volcano plot in Fig. 6D. Noteworthy, specific upregulated transcripts, including *B2m*, *Saa1* and *Saa2*, have been associated in the post-partum human mammary gland with a condition of high breast tumor risk (Asztalos et al., 2010; Borges et al., 2020; Jindal et al., 2020). In summary, this analysis suggests that during lactation abnormal RET levels drive a transcriptomic signature that is enriched in Stat-driven genes related to the tumor-promoting inflammatory-phase response at the onset of involution.

**Table 2.**
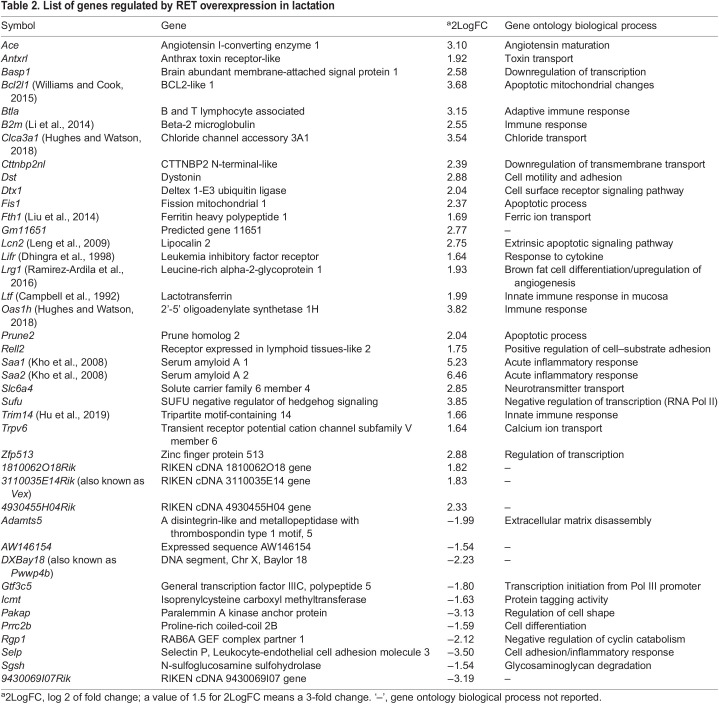
List of genes regulated by RET overexpression in lactation

## DISCUSSION

We show here that RET is expressed during lactation and is normally downregulated during the transition to involution. We demonstrate that the reduction in RET at involution onset is due to local signals induced by lactation cessation (Fig. 2). The specific signals responding to milk stasis resulting in decreased RET levels in involution remain unknown, but a systemic decrease in circulating lactogenic hormones is not involved. Moreover, we show that abnormal expression of RET in the mammary epithelium during the transition to involution causes a defect in the post-lactation process, leading to cancer predisposition. Mechanistically, our results suggest that a RET-driven Stat3-mediated acute inflammatory signature is a part of the process.

Using Ret/MTB mice, we have previously reported that overexpression of WT *Ret* gene is oncogenic and that multiparous females exhibit higher mammary tumor incidence than nulliparous females (Gattelli et al., 2018). In the present work, we analyzed earlier steps in tumor development using Ret/MTB mice. We show that forced RET overexpression in lactation leads to an increase in epithelial cell proliferation (Fig. 6C), which likely contributes to the higher tumor incidence of the multiparous females. Indeed, we identified more neoplastic lesions in the Ret/MTB glands from parous mice following 2 months of involution (Post-I) compared to control glands from nulliparous mice (5.94±1.52 versus 2.60±1.39, respectively) (Table 1), indicating that the involution environment supports RET-induced tumorigenesis. On that note, our results are in line with seminal studies in mammary tumor models demonstrating that involution has pro-tumorigenic effects that are associated with the late phase of wound healing (Lyons et al., 2011; Martinson et al., 2015; O'Brien et al., 2010; Pennock et al., 2018).

Overexpression of RET from lactation through early involution leads to a more rapid appearance of involution markers. Interestingly, mouse models investigating effects of known breast oncogenes often showed alterations in normal involution (Radisky and Hartmann, 2009), similar to what we show for the Ret/MTB model. In most cases, suppression of cell death delays involution and facilitates tumor progression, e.g. in mice expressing Akt1 (Hutchinson et al., 2001), ERBB2 (Castillo-Lluva et al., 2015) or Bcl2 (Jäger et al., 1997). In contrast, mice constitutively expressing Myc exhibited accelerated involution that correlated with an increased incidence of cancer (Jäger et al., 1997; Stewart et al., 1984), as we observe with the Ret/MTB model. The reason for premature involution in Myc-expressing glands remains unclear (Radisky and Hartmann, 2009). For RET, it is possible that the pro-inflammatory signals stimulated by RET-triggered alterations in the mammary epithelium accelerate involution. In fact, in thyroid cancer, RET signaling has been related to activation of inflammatory programs (Guarino et al., 2010).

The acute inflammatory signature observed during early involution, in which Stat3 has a pivotal role, has also been described as tumor promotional (Pensa et al., 2009). Indeed, we demonstrated that Post-I RET-expressing hyperplastic glands show increased Stat3 activity compared to RET-expressing hyperplastic glands from virgin females (Fig. 4E). Stat3 has dual functions: it has an essential role in initiating cell death in involution, but an oncogenic role when chronically activated in the mammary epithelium (Dechow et al., 2004; Pensa et al., 2009). Taken together, these results indicate that RET activation in an involuting pro-inflammatory environment propels mammary epithelial cells into a neoplastic state. We propose that Stat3 activation downstream of persistent RET signaling unleashes apoptosis, accelerating the involution process, and could simultaneously increase inflammation, culminating in chronic Stat3 activation-promoting cancer (Figs S3 and S4). Another possibility is that the Akt signaling upregulation driven by RET expression (Fig. 6B) also contributes to oncogenic stress signals, which leads to the presence of apoptotic cells. Although RET function in normal mammary gland is not fully elucidated, it might potentially contribute to survival signals in lactating mammary cells. In the future, this could be assessed following conditional deletion of *Ret* in the mammary epithelium.

Lactation is an important factor affecting breast cancer incidence in both young and post-menopausal females. Lactation has significant protective effects against specific breast cancer subtypes (Palmer et al., 2014), and, overall, breastfeeding reduces cancer risk in Caucasian females (Amant et al., 2013). Therefore, understanding how lactation impacts breast cancer risk and whether it counters adverse effects of involution is critical. Our studies reveal that RET overexpression in lactation upregulates genes that are pro-tumorigenic such as *Bcl2l1* or *Lcn2* (Leng et al., 2009; Williams and Cook, 2015), but also genes associated with early involution such as *Clca3a1* or *Oas1h* (Clarkson et al., 2004; Stein et al., 2007) (Fig. 6). The highest transcriptionally increased genes are *Saa1* and *Saa2*, which are direct Stat3-target genes normally increased at involution onset (Hughes and Watson, 2018). It is worth highlighting that the transcriptome of RET-expressing lactating glands shares genes (Asztalos et al., 2010; Jindal et al., 2020) identified in post-partum breast tissue collected during the first 5 year period, a time frame in which there is an increased tumor risk (Borges et al., 2020). Thus, our analysis indicates that, in mammary epithelium, abnormal RET expression resembles the early involution, characterized by an acute-phase inflammatory response-like signature more than the wound-healing-like phase at later stages. This conclusion is strengthened by our observation that, in the Ret/MTB glands, there are no differences in macrophage cell influx, an essential component of the second phase of involution, in comparison to controls (Fig. S4B). Despite our findings and other research insights in mammary cancer models (Gattelli et al., 2020), the connection between RET and inflammation remains understudied. Further investigation needs to be performed to determine the impact of RET-expressing mammary cells on the immune/inflammatory population.

In summary, we have discovered that, during mammary gland development, RET is downregulated early in involution and its overexpression during this period causes defects in the post-lactation transition, ultimately leading to an increased cancer predisposition by a mechanism involving persistent Stat3 activation. We have observed accelerated involution after forced RET expression, which is interesting because RET is usually downregulated during normal involution. RET-induced perturbation at the post-partum stage results in a pro-tumorigenic phenotype, which might be due to RET-driven changes in mammary epithelium specifically susceptible to oncogenic transformation in concert with a pro-inflammatory microenvironment during early involution. RET is overexpressed in ∼50% of breast tumors and has been suggested as an alternative therapeutic target, particularly in light of the recent advances in novel RET inhibitors (Belli et al., 2020; Gattelli et al., 2020; Paratala et al., 2018). Our analysis using mouse models offers new insights, suggesting that RET expression levels could be a biomarker of post-lactation-associated breast tumors.

## MATERIALS AND METHODS

### Mice

WT FVB/N (Taconic) mice were used in specific experiments. Female mice were mated between 8 and 12 weeks of age. For timed pregnancies (15-19 days of pregnancy), adult female mice were mated, scored by the presence of vaginal plugs and confirmed by the examination of embryos at the time of mammary gland collection. For involution studies, dams were allowed to nurse litters (normalized to seven to ten pups) for 8-12 days, and then pups were separated from dams to initiate forced involution. Glands were harvested from virgin females (V), pregnant females (P), dams lactating for 5 days (L5), 10 days (L10) or 15 days (L15), or from dams involuting 12 h (I12), 48 h (I48) or 72 h (I72) following pup removal. At least three mice per group were analyzed for each developmental stage. Ret/MTB transgenic mice (Gattelli et al., 2018) were originally generated in the Friedrich Miescher Institute for Biomedical Research (FMI), Basel, Switzerland. This transgenic strain was shipped, maintained and established as a colony in the animal facility from the Institute of Physiology, Molecular Biology and Neuroscience (IFIBYNE), University of Buenos Aires (UBA), Buenos Aires, Argentina. Breeding and the genotypes of offspring were determined by PCR amplification of ear DNA as described (Gattelli et al., 2018). Transgenic and control mice were administered a control diet (Kliba Nafag #3302.PM.V20, irradiation >25 Gy), one enriched with 200 mg/kg DOX in the chow (Sigma by Chow Manufactures) or received 2 g/l DOX in drinking water (Jenner Lafarvet). Ret/MTB adult female mice were monitored for luciferase activity after 3-7 days of DOX or control diet following published procedures (Gattelli et al., 2018). For transgenic animals, developmental stages, virgin (V), lactation (L) and involution (I), were considered as described for WT mice. Specifically, a post-involution stage (Post-I) was defined as a post-lactation 2 month-regressed gland after pup removal. For that, at day 10 of lactation, pups were removed (considering this the first day of involution), treatments were initiated, and, 2 months later, Post-I glands under DOX or control diet were extracted and stored for analysis. For all mouse experiments, mouse numbers and independent biological experiments are listed in the figures and figure legends. Transgenic and non-transgenic mice were maintained in a specific pathogen-free facility, at constant temperature and humidity with a 12 h light cycle. Animals were allowed food and water *ad libitum*. Mice were housed under hygienic conditions according to the Swiss and Argentinian guidelines governing animal experimentation. All treatments and euthanasia protocols used in these studies were reviewed and approved by the Institutional Animal Care and Use Committee (IACUC).

### Quantitative real-time PCR (qRT-PCR) and RT-PCR

For each stage of mammary gland development, an intact inguinal fourth (#4) mammary gland was removed and homogenized for 15 s using Ultraturrax T25 (Ika Labortechnik Staufen) in RNA-PrepZOL (Inbio Highway), according to the instructions. For HC11 cell cultures, total RNA was extracted. Two micrograms of total RNA were used for reverse transcription according to the M-MLV reverse transcriptase protocol (Promega). RT-PCR was performed in a Bio-Rad instrument using standard reaction mix containing DNA polymerase (K1001 INBIO Highway) up to 40 cycles of amplification. The amplification product was analyzed by electrophoresis in agarose gel (RU1007 LE-Agarose 1200). The program was as follows: 94°C for 2 min, 40 cycles of 94°C for 15 s, 60°C for 20 s, 72°C for 1 min/Kb, plus a final extension step of 72°C for 4 min. qRT-PCR was performed on the StepOne Plus instrument (Applied Biosystems) using SYBR Green (Roche) in the reaction mix. For qRT-PCR, calibration curves were performed for each specific primer pair and used in the calculation for quantification. The program was as follows: 95°C for 5 min, 40 cycles of 95°C for 15 s, 60°C for 20 s, 72°C for 20 s, plus standard program for melting curves. The following primers (synthesized by Invitrogen or Macrogen) were used: *Gdnf* Fw, 5′-AGGAGGAACTGATTTTTAGGTAC-3′; *Gdnf* Rv, 5′-TGCCCTACTTTGTCACTCAC-3′; *Nrtn* Fw, 5′-CTGGAAGGCAGCGGCCC-3′; *Nrtn* Rv, 5′-CCCTGGAGCAGAGCGCG-3′; *Gfra1* Fw, 5′-CTGTGTGCTCCTATGAAGAACGA-3′; *Gfra1* Rv, 5′-TTGCTGCAATCGCACCACGGC-3′; *Gfra2* Fw, 5′-GTATACCTACCGCATGCTCTTC-3′; *Gfra2* Rv, 5′-GGGCTTCTCTTTGTCCTCATAG-3′; *Actb* Fw, 5′-TGCGTGACATCAAAGAGAAG-3′; *Actb* Rv, 5′-TGCGTGACATCAAAGAGAAG-3′; *Ret* Fw, 5′-GGACATCCATTACTGAAGAAGTA-3′; *Ret* Rv, 5′-AGTCCTGGGGGCAAATGTTGGCA-3′.

### Morphological and histological analysis

For whole-mount analysis, inguinal fourth (#4) mammary glands were stretched onto a glass slide and fixed with Carnoy's fixative (ethanol/chloroform/glacial acetic acid at proportions of 6:3:1, respectively) for 24 h at room temperature. Then, they were rehydrated with a graded series of ethanol solutions, washed for 5 min in distilled water and stained overnight in alum carmine solution. The following day, the mammary glands were dehydrated with a graded series of ethanol solutions. Mammary glands were cleared in xylene until the fat had been sufficiently removed from the glands. For histopathological studies, mammary glands were fixed in 4% paraformaldehyde, stored in 70% ethanol, embedded in paraffin, sectioned and stained with H&E. For analysis of neoplastic lesions, the inguinal #4 as well as the second (#2) mammary glands were dissected post-euthanasia for each animal group. For quantification of the neoplastic lesions, H&E-stained slides of mammary glands were observed with an optic microscope (40×). Neoplastic lesions were named as MIN and defined as an accumulation of more than 200 neoplastic cells without tissue organization. The number of MIN was calculated by examination of three different slides (4 µm) corresponding to three distinct areas of the gland obtained by serial intervals (separated by at least 40 µm). For this analysis, two different mammary glands (#4 and #2) from the same mouse were used, coming from independent animals, in the experimental and control groups.

### IHC staining

In specific cases, automated chromogenic IHC for different markers was carried out in paraffin sections using the Ventana DiscoveryXT instrument (Roche Diagnostics) and the following antibodies: anti-CC3 (#9661, Cell Signaling Technology, 1:100, DAB Map XT protocol), anti-RET (#14556, Cell Signaling Technology, 1:150, DAB Map XT protocol) and anti-p-Y705Stat3 (#9145, Cell Signaling Technology, 1:100, DAB Map XT protocol). Manual IHC was performed in some cases. After sample dewaxing and rehydration, heat-induced epitope retrieval was performed with 10 mM sodium citrate pH 6.0 for 10 min at sub-boiling temperature. Sections were exposed for 10 min to 3% H_2_O_2_ and, after washing, blocked for 1 h in 2.5% bovine serum albumin (BSA). The samples were incubated overnight at 4°C with the following antibodies diluted in 2.5% BSA: anti-perilipin-1 (#3470, Cell Signaling Technology, 1:200), anti-p-Y705Stat3 (#9145, Cell Signaling Technology, 1:100), anti-p-S10H3 (#9701, Cell Signaling Technology, 1:200), anti-β-casein (sc-166530, Santa Cruz Biotechnology, 1:1000), anti-F4/80 (#CI:A3-1, Serotec, 1:50) and anti-S100A9 (#73425, Cell Signaling Technology, 1:800). The next day, after washing, the samples were incubated for 1 h with an anti-rabbit biotinylated antibody, diluted 1:200 in 2.5% BSA. Next, samples were washed and incubated with the ABC reagent (PK-6101, Vector Laboratories) for 30 min. Finally, the sections were washed and stained with DAB and Hematoxylin as a counterstain. For p-Y705Stat3 and CC3-staining, pictures (400× and 200×, respectively) covering the whole area of the gland were taken. Quantification was done using ImageJ software and expressed as stained area fraction/field±s.d. For perilipin-1 staining, pictures (40×) covering the whole area of the gland were taken. Quantification was done by measuring the percentage of positively stained area/field±s.d. For p-S10H3b and in some cases for p-Y705Stat3, pictures (400×) of five random areas of the gland were taken. For each staining, quantification was done by counting and expressing as the number of positive cells/field±s.d. or by counting positive and negative epithelial cells and expressing as a percentage (%) of positive cells/field±s.d., respectively. In the figure legends, the magnification of the pictures is indicated by scale bars.

### Protein extraction and WB

Whole mammary glands were homogenized for 15 s in RIPA buffer containing a protease and phosphatase inhibitor cocktail (25 mM β-Glycerophosphate, 25 mM NaF, 1 mM DTT, 1 mM PMSF and 2 mM orthovanadate), using Ultraturrax T25 (Ika Labortechnik Staufen). Following 30 min on ice, lysates were cleared by centrifugation at 20,000 ***g***, and the supernatants were collected and stored at −80°C. For cell cultures, cells from the monolayer were lysed in a buffer containing 50 mM Hepes (pH 7.4), 150 mM NaCl, 25 mM β-Glycerophosphate, 25 mM NaF, 5 mM EGTA, 15 mM PPI, 1% NP40 and 1 mM DTT, containing the inhibitors, 1 mM PMSF, 10 μg/ml aprotinin, 10 μg/ml leupeptin, 2 mM sodium orthovanadate and 10 mM sodium molybdate. Before freezing, an aliquot of lysate was diluted at 1:10 in PBS 1× for determination of the protein concentration with a Bradford assay using BSA as standard. Extracts of total protein (40-60 µg) were separated using sodium dodecyl sulfate polyacrylamide gel electrophoresis (SDS-PAGE) and subsequently transferred onto PVDF blotting membrane by a semi-dry transfer (Bio-Rad). The membranes were blocked with 5% Teleostean gelatin (#G7765, Sigma-Aldrich) in PBS-T (pH 7.6; 0.5% Tween 20) for 1 h at room temperature. Subsequently, the membranes were incubated overnight at 4°C with the primary antibody diluted in 5% Teleostean gelatin in PBS-T. The following primary antibodies were used for overnight incubations: anti-RET (#3223, Cell Signaling Technology, 1:1000), anti-p-Y1062RET (sc-2052, Santa Cruz Biotechnology, 1:1000), anti-p-Y694Stat5 (#4322, Cell Signaling Technology, 1:5000), anti-p-S779Stat5 (laboratory made, 1:1000), anti-Stat5 (#9363, Cell Signaling Technology, 1:5000), anti-p-Y705Stat3 (#9131, Cell Signaling Technology, 1:5000), anti-Stat3 (#610190, Transduction Laboratories, 1:5000), anti-p-T202/Y204Erk (#9101, Cell Signaling Technology, 1:5000), anti-Erk (#9102, Cell Signaling Technology, 1:5000), anti-p-S473Akt (#4060, Cell Signaling Technology, 1:5000), anti-Akt (#9272, Cell Signaling Technology, 1:5000) and anti-α-tubulin (MS-581-P1, Neomarkers, 1:10,000). The following day, the primary antibodies were removed, and the membranes were washed in PBS-T. Subsequently, the secondary horseradish peroxidase (HRP)-conjugated antibody (Amersham, GE Healthcare) anti-rabbit (#NA934, 1:10,000) and/or anti-mouse (#NA931, 1:10,000) was added and incubated for 1 h at room temperature. Finally, the membranes were visualized using the ECL Western Blotting System (Amersham, GE Healthcare) and CL-XPosure films (#34089, Thermo Fisher Scientific) or medical X-ray films Ortho CP-GU M (Agfa). For RET protein level quantifications, RET as well as tubulin bands on WBs were quantified using ImageJ. Lysates corresponding to mammary samples from independent animals at each developmental stage were used. Results were expressed as ratios, RET relative to tubulin.

### RNA-sequencing and enrichment analysis

Mammary gland tissues were processed to obtain RNA from DOX-induced Ret/MTB, Ret/− and MTB/− 10 day-lactating mice, and Ret/− virgin mice. Total RNA was obtained using an RNeasy Mini Kit (Qiagen). After quality control, sequencing libraries were prepared from 200 ng total RNA using a TruSeq Stranded mRNA Library Preparation Kit (Illumina). Libraries were pooled and sequenced on an Illumina HiSeq2500. HCS 2.2.58 was used with default parameters for base calling and bcl2fastq 1.8.4 (Illumina) for de-multiplexing and generating fastq files. Raw reads were preprocessed using the R(v3.3.0)/Bioconductor package QuasR(1.12.0). Reads with more than 2Ns were removed with the function preprocess reads. The remaining reads were aligned to mm10 using the qAlign with splicedAlignments=TRUE and default parameters. The QuasR function qCount was used to extract a table with raw read counts per gene for all Entrez genes for each sample. The data have been submitted to the NCBI and assigned the BioProject ID PRJNA 722027. Pathway analysis was performed with GSEA (http://software.broadinstitute.org/gsea) on the complete gene expression profile for each sample. The table was generated through differentially expressed gene values. The volcano plot was constructed from complete gene expression data using R.

### Cell culture

HC11 cells (Hynes laboratory origin) were grown in RPMI 1640 (Gibco) supplemented with 10% FBS, 100 IU/ml penicillin, 100 μg/ml streptomycin, 4 mM L-glutamine, 10 ng/ml EGF and 5 μg/ml insulin (Sigma-Aldrich). HC11 competent cultures (non-proliferating) were obtained as published (Williams et al., 2009) by allowing cells to reach confluency and maintaining cultures for 2 days in 2% FBS after EGF and glutamine withdrawal. HC11 cultures [serum-starved (RPMI+2% FBS)] were stimulated either with ligand (GDNF) at 10 ng/ml or with vehicle (H_2_O) and cells were collected 5, 15 and 30 min post-stimulation. RET-ligand GDNF (#450-10, PeproTech) was re-suspended according to the manufacturer's instructions, aliquoted and stored at −80°C, and the aliquots were only thawed twice.

### Statistical analysis

Data were plotted using Excel or GraphPad software. Imaging quantification was performed using ImageJ. Statistical analysis of the results was determined using the appropriate GraphPad software test. Before selecting a statistical test, normality was assessed by the Shapiro–Wilk test to ensure validity of the parametric tests. When the generated data were found to deviate significantly from a normal distribution, we used non-parametric tests. Otherwise, we used two-sided two-sample *t*-tests to compare measurements between treatment and control groups, and, when several variables were simultaneously observed, the corresponding multivariate ANOVA test was performed. The results were considered statistically significant if the *P*-value of a statistical hypothesis test was smaller than the significance level of 0.05. All animal studies were replicated at least twice and representative or pooled data are shown. The number of required mice per replicate group was estimated assuming normality and aiming for a power of 80% using the statistical test and a significance level of α=0.05. We chose the level of statistical significance to be α=0.05. The means and s.d. were estimated based on a pilot experiment. For the overall design of the experiments, we used the randomization method to allocate the experimental groups. Accordingly, as previously experienced, we observed no animal dropout or death following the administration of DOX to induce the overexpression of *Ret* gene (Gattelli et al., 2018). We did not omit data points for *in vivo* experiments. Outlier robust rank-based nonparametric test was performed when outliers were suspected.

## Supplementary Material

Supplementary information

## References

[DMM049286C1] Abell, K., Bilancio, A., Clarkson, R. W. E., Tiffen, P. G., Altaparmakov, A. I., Burdon, T. G., Asano, T., Vanhaesebroeck, B. and Watson, C. J. (2005). Stat3-induced apoptosis requires a molecular switch in PI(3)K subunit composition. *Nat. Cell Biol.* 7, 392-398. 10.1038/ncb1242 15793565

[DMM049286C2] Akhtar, N., Li, W., Mironov, A. and Streuli, C. H. (2016). Rac1 controls both the secretory function of the mammary gland and its remodeling for successive gestations. *Dev. Cell* 38, 522-535. 10.1016/j.devcel.2016.08.005 27623383 PMC5022528

[DMM049286C3] Amant, F., Von Minckwitz, G., Han, S. N., Bontenbal, M., Ring, A. E., Giermek, J., Wildiers, H., Fehm, T., Linn, S. C., Schlehe, B. et al. (2013). Prognosis of women with primary breast cancer diagnosed during pregnancy: Results from an international collaborative study. *J. Clin. Oncol.* 31, 2532-2539. 10.1200/JCO.2012.45.6335 23610117

[DMM049286C4] Asztalos, S., Gann, P. H., Hayes, M. K., Nonn, L., Beam, C. A., Dai, Y., Wiley, E. L. and Tonetti, D. A. (2010). Gene expression patterns in the human breast after pregnancy. *Cancer Prev. Res.* 3, 301-311. 10.1158/1940-6207.CAPR-09-0069 20179293

[DMM049286C5] Bambhroliya, A., Van Wyhe, R. D., Kumar, S., Debeb, B. G., Reddy, J. P., Van Laere, S., El-Zein, R., Rao, A. and Woodward, W. A. (2018). Gene set analysis of post-lactational mammary gland involution gene signatures in inflammatory and triple-negative breast cancer. *PLoS ONE* 13, e0192689. 10.1371/journal.pone.0192689 29617367 PMC5884491

[DMM049286C6] Baxter, F. O., Neoh, K. and Tevendale, M. C. (2007). The beginning of the end: death signaling in early involution. *J. Mammary Gland Biol. Neoplasia* 12, 3-13. 10.1007/s10911-007-9033-9 17340185

[DMM049286C7] Belli, C., Anand, S., Gainor, J. F., Penault-Llorca, F., Subbiah, V., Drilon, A., Andrè, F. and Curigliano, G. (2020). Progresses toward precision medicine in RET -altered solid tumors. *Clin. Cancer Res.* 26, 6102-6111. 10.1158/1078-0432.CCR-20-1587 32665298

[DMM049286C8] Borges, V. F., Lyons, T. R., Germain, D. and Schedin, P. (2020). Postpartum involution and cancer: an opportunity for targeted breast cancer prevention and treatments? *Cancer Res.* 80, 1790-1798. 10.1158/0008-5472.CAN-19-3448 32075799 PMC8285071

[DMM049286C9] Boulay, A., Breuleux, M., Stephan, C., Fux, C., Brisken, C., Fiche, M., Wartmann, M., Stumm, M., Lane, H. A. and Hynes, N. E. (2008). The ret receptor tyrosine kinase pathway functionally interacts with the ERa pathway in breast cancer. *Cancer Res.* 68, 3743-3751. 10.1158/0008-5472.CAN-07-5100 18483257

[DMM049286C10] Bromberg, J. and Darnell, J. E. (2000). The role of STATs in transcriptional control and their impact on cellular function. *Oncogene* 19, 2468-2473. 10.1038/sj.onc.1203476 10851045

[DMM049286C11] Callihan, E. B., Gao, D., Jindal, S., Lyons, T. R., Manthey, E., Edgerton, S., Urquhart, A., Schedin, P. and Borges, V. F. (2013). Postpartum diagnosis demonstrates a high risk for metastasis and merits an expanded definition of pregnancy-associated breast cancer. *Breast Cancer Res. Treat.* 138, 549-559. 10.1007/s10549-013-2437-x 23430224 PMC3608871

[DMM049286C73] Campbell, T., Skilton, R. A., Coombes, R. C., Shousha, S., Graham, M. D. and Luqmani, Y. A. (1992). Isolation of a lactoferrin cdna clone and its expression in human breast cancer. *Br. J. Cancer* 65, 19-26. 10.1038/bjc.1992.4 1733438 PMC1977368

[DMM049286C12] Castillo-Lluva, S., Hontecillas-Prieto, L., Blanco-Gómez, A., Del Mar Sáez-Freire, M., García-Cenador, B., García-Criado, J., Pérez-Andrés, M., Orfao, A., Cañamero, M., Mao, J. H. et al. (2015). A new role of SNAI2 in postlactational involution of the mammary gland links it to luminal breast cancer development. *Oncogene* 34, 4777-4790. 10.1038/onc.2015.224 26096931 PMC4560637

[DMM049286C13] Chapman, R. S., Lourenco, P. C., Tonner, E., Flint, D. J., Selbert, S., Takeda, K., Akira, S., Clarke, A. R. and Watson, C. J. (1999). Suppression of epithelial apoptosis and delayed mammary gland involution in mice with a conditional knockout of Stat3. *Genes Dev.* 13, 2604-2616. 10.1101/gad.13.19.2604 10521404 PMC317074

[DMM049286C14] Clarkson, R. W. E., Wayland, M. T., Lee, J., Freeman, T. and Watson, C. J. (2004). Gene expression profiling of mammary gland development reveals putative roles for death receptors and immune mediators in post-lactational regression. *Breast Cancer Res.* 6, R92-R109. 10.1186/bcr754 14979921 PMC400653

[DMM049286C15] Clarkson, R. W. E., Boland, M. P., Kritikou, E. A., Lee, J. M., Freeman, T. C., Tiffen, P. G. and Watson, C. J. (2006). The genes induced by signal transducer and activators of transcription (STAT)3 and STAT5 in mammary epithelial cells define the roles of these STATs in mammary development. *Mol. Endocrinol.* 20, 675-685. 10.1210/me.2005-0392 16293640

[DMM049286C16] Creamer, B. A., Sakamoto, K., Schmidt, J. W., Triplett, A. A., Moriggl, R. and Wagner, K.-U. (2010). Stat5 promotes survival of mammary epithelial cells through transcriptional activation of a distinct promoter in Akt1. *Mol. Cell. Biol.* 30, 2957-2970. 10.1128/MCB.00851-09 20385773 PMC2876679

[DMM049286C17] De Groot, J. W. B., Links, T. P., Plukker, J. T. M., Lips, C. J. M. and Hofstra, R. M. W. (2006). RET as a diagnostic and therapeutic target in sporadic and hereditary endocrine tumors. *Endocr. Rev.* 27, 535-560. 10.1210/er.2006-0017 16849421

[DMM049286C18] Debnath, J. (2008). Detachment-induced autophagy during anoikis and lumen formation in epithelial acini. *Autophagy* 4, 351-353. 10.4161/auto.5523 18196957

[DMM049286C19] Dechow, T. N., Pedranzini, L., Leitch, A., Leslie, K., Gerald, W. L., Linkov, I. and Bromberg, J. F. (2004). Requirement of matrix metalloproteinase-9 for the transformation of human mammary epithelial cells by Stat3-C. *Proc. Natl. Acad. Sci. U. S. A.* 101, 10602-10607. 10.1073/pnas.0404100101 15249664 PMC489981

[DMM049286C76] Dhingra, K., Sahin, A., Emami, K., Hortobagyi, G. N. and Estrov, Z. (1998). Expression of leukemia inhibitory factor and its receptor in breast cancer: A potential autocrine and paracrine growth regulatory mechanism. *Breast Cancer Res. Treat*. 48, 165-174. 10.1023/a:1005942923757 9596488

[DMM049286C20] Drilon, A., Hu, Z. I., Lai, G. G. Y. and Tan, D. S. W. (2018). Targeting ret-driven cancers: Lessons from evolving preclinical and clinical landscapes. *Nat. Rev. Clin. Oncol.* 15, 151-167. 10.1038/nrclinonc.2017.175 29134959 PMC7938338

[DMM049286C21] Fornetti, J., Martinson, H. A., Betts, C. B., Lyons, T. R., Jindal, S., Guo, Q., Coussens, L. M., Borges, V. F. and Schedin, P. (2014). Mammary gland involution as an immunotherapeutic target for postpartum breast cancer. *J. Mammary Gland Biol. Neoplasia* 19, 213-228. 10.1007/s10911-014-9322-z 24952477 PMC4363120

[DMM049286C22] Gattelli, A., Nalvarte, I., Boulay, A., Roloff, T. C., Schreiber, M., Carragher, N., Macleod, K. K., Schlederer, M., Lienhard, S., Kenner, L. et al. (2013). Ret inhibition decreases growth and metastatic potential of estrogen receptor positive breast cancer cells. *EMBO Mol. Med.* 5, 1335-1350. 10.1002/emmm.201302625 23868506 PMC3799490

[DMM049286C23] Gattelli, A., García Solá, M. E., Roloff, T. C., Cardiff, R. D., Kordon, E. C., Chodosh, L. A. and Hynes, N. E. (2018). Chronic expression of wild-type Ret receptor in the mammary gland induces luminal tumors that are sensitive to Ret inhibition. *Oncogene* 37, 4046-4054. 10.1038/s41388-018-0235-y 29695833

[DMM049286C24] Gattelli, A., Hynes, N. E., Schor, I. E. and Vallone, S. A. (2020). Ret receptor has distinct alterations and functions in breast cancer. *J. Mammary Gland Biol. Neoplasia* 25, 13-26. 10.1007/s10911-020-09445-4 32080788

[DMM049286C25] Grinman, D. Y., Careaga, V. P., Wellberg, E. A., Dansey, M. V., Kordon, E. C., Anderson, S. M., Maier, M. S., Burton, G., MacLean, P. S., Rudolph, M. C. et al. (2019). Liver X receptor-α activation enhances cholesterol secretion in lactating mammary epithelium. *Am. J. Physiol. Metab.* 316, E1136-E1145. 10.1152/ajpendo.00548.2018 PMC6620573 30964702

[DMM049286C26] Guarino, V., Castellone, M. D., Avilla, E. and Melillo, R. M. (2010). Thyroid cancer and inflammation. *Mol. Cell. Endocrinol.* 321, 94-102. 10.1016/j.mce.2009.10.003 19835928

[DMM049286C79] Gunther, E. J., Belka, G. K., Wertheim, G. B. W., Wang, J., Hartman, J. L., Boxer, R. B. and Chodosh, L. A. (2002). A novel doxycycline-inducible system for the transgenic analysis of mammary gland biology. *FASEB J.* 16, 283-292. 10.1096/fj.01-0551com 11874978

[DMM049286C27] Haricharan, S. and Li, Y. (2014). STAT signaling in mammary gland differentiation, cell survival and tumorigenesis. *Mol. Cell. Endocrinol.* 382, 560-569. 10.1016/j.mce.2013.03.014 23541951 PMC3748257

[DMM049286C72] Hu, G., Pen, W. and Wang, M. (2019). TRIM14 promotes breast cancer cell proliferation by inhibiting apoptosis. *Oncol. Res.* 27, 439-447. 10.3727/096504018X15214994641786 29562956 PMC7848417

[DMM049286C28] Hughes, K. and Watson, C. J. (2018). The multifaceted role of STAT3 in mammary gland involution and breast cancer. *Int. J. Mol. Sci.* 19, 1695. 10.3390/ijms19061695 PMC6032292 29875329

[DMM049286C29] Hughes, K., Wickenden, J. A., Allen, J. E. and Watson, C. J. (2012). Conditional deletion of Stat3 in mammary epithelium impairs the acute phase response and modulates immune cell numbers during post-lactational regression. *J. Pathol.* 227, 106-117. 10.1002/path.3961 22081431 PMC3477635

[DMM049286C30] Hume, R. D., Pensa, S., Brown, E. J., Kreuzaler, P. A., Hitchcock, J., Husmann, A., Campbell, J. J., Lloyd-Thomas, A. O., Cameron, R. E. and Watson, C. J. (2018). Tumour cell invasiveness and response to chemotherapeutics in adipocyte invested 3D engineered anisotropic collagen scaffolds. *Sci. Rep.* 8, 12658. 10.1038/s41598-018-30107-3 30139956 PMC6107500

[DMM049286C31] Humphreys, R. C., Bierie, B., Zhao, L., Raz, R., Levy, D. and Hennighausen, L. (2002). Deletion of Stat3 blocks mammary gland involution and extends functional competence of the secretory epithelium in the absence of lactogenic stimuli. *Endocrinology* 143, 3641-3650. 10.1210/en.2002-220224 12193580

[DMM049286C32] Hutchinson, J., Jin, J., Cardiff, R. D., Woodgett, J. R. and Muller, W. J. (2001). Activation of Akt (Protein Kinase B) in mammary epithelium provides a critical cell survival signal required for tumor progression. *Mol. Cell. Biol.* 21, 2203-2212. 10.1128/MCB.21.6.2203-2212.2001 11238953 PMC86854

[DMM049286C33] Hynes, N. E. and Watson, C. J. (2010). Mammary gland growth factors: roles in normal development and in cancer. *Cold Spring Harb. Perspect. Biol.* 2, a003186. 10.1101/cshperspect.a003186 20554705 PMC2908768

[DMM049286C34] Ibáñez, C. F. (2013). Structure and physiology of the RET receptor tyrosine kinase. *Cold Spring Harb. Perspect. Biol.* 5, a009134. 10.1101/cshperspect.a009134 23378586 PMC3552510

[DMM049286C35] Jäger, R., Herzer, U., Schenkel, J. and Weiher, H. (1997). Overexpression of Bcl-2 inhibits alveolar cell apoptosis during involution and accelerates c-myc-induced tumorigenesis of the mammary gland in transgenic mice. *Oncogene* 15, 1787-1795. 10.1038/sj.onc.1201353 9362445

[DMM049286C36] Jindal, S., Narasimhan, J., Borges, V. F. and Schedin, P. (2020). Characterization of weaning-induced breast involution in women: implications for young women's breast cancer. *npj Breast Cancer* 6, 55. 10.1038/s41523-020-00196-3 33083533 PMC7568540

[DMM049286C74] Kho, Y., Kim, S., Yoon, B., Moon, J.-H. and Kim, B. (2008). Induction of serum amyloid A genes Is associated with growth and apoptosis of HC11 mammary epithelial cells. *Biotechnol. Biochem*. 72, 70-81. 10.1271/bbb.70374 18175929

[DMM049286C37] Kreuzaler, P. A., Staniszewska, A. D., Li, W., Omidvar, N., Kedjouar, B., Turkson, J., Poli, V., Flavell, R. A., Clarkson, R. W. E. and Watson, C. J. (2011). Stat3 controls lysosomal-mediated cell death in vivo. *Nat. Cell Biol.* 13, 303-309. 10.1038/ncb2171 21336304

[DMM049286C38] Leng, X., Ding, T., Lin, H., Wang, Y., Hu, L., Hu, J., Feig, B., Zhang, W., Pusztai, L., Symmans, W. F. et al. (2009). Inhibition of lipocalin 2 impairs breast tumorigenesis and metastasis. *Cancer Res.* 69, 8579-8584. 10.1158/0008-5472.CAN-09-1934 19887608

[DMM049286C39] Li, M., Liu, X., Ribinson, G., Bar-Peled, U., Wagner, K. U., Young, W. S., Hennighausen, L. and Furth, P. A. (1997). Mammary-derived signals activate programmed cell death during the first stage of mammary gland involution. *Proc. Natl. Acad. Sci. U. S. A.* 94, 3425-3430. 10.1073/pnas.94.7.3425 9096410 PMC20386

[DMM049286C40] Li, G., Robinson, G. W., Lesche, R., Martinez-Diaz, H., Jiang, Z., Rozengurt, N., Wagner, K.-U., Wu, D.-C., Lane, T. F., Liu, X. et al. (2002). Conditional loss of PTEN leads to precocious development and neoplasia in the mammary gland. *Development* 129, 4159-4170. 10.1242/dev.129.17.4159 12163417

[DMM049286C75] Li, K., Du, H., Lian, X., Yang, S., Chai, D., Wang, C., Yang, R. and Chen, X. (2014). Characterization of β2-microglobulin expression in different types of breast cancer. *BMC Cancer* 14, 750. 10.1186/1471-2407-14-750 25292288 PMC4197271

[DMM049286C41] Liberzon, A., Birger, C., Thorvaldsdóttir, H., Ghandi, M., Mesirov, J. P. and Tamayo, P. (2015). The molecular signatures database hallmark gene set collection. *Cell Syst.* 1, 417-425. 10.1016/j.cels.2015.12.004 26771021 PMC4707969

[DMM049286C42] Lima, S. M., Kehm, R. D., Swett, K., Gonsalves, L. and Terry, M. B. (2020). Trends in parity and breast cancer incidence in us women younger than 40 years from 1935 to 2015. *JAMA Netw. Open* 3, e200929. 10.1001/jamanetworkopen.2020.0929 32167569 PMC7070232

[DMM049286C43] Liu, X., Robinson, G. W., Wagner, K. U., Garrett, L., Wynshaw-Boris, A. and Hennighausen, L. (1997). Stat5a is mandatory for adult mammary gland development and lactogenesis. *Genes Dev.* 11, 179-186. 10.1101/gad.11.2.179 9009201

[DMM049286C77] Liu, N. Q., De Marchi, T., Timmermans, A. M., Beekhof, R., Trapman-Jansen, A. M. A. C., Foekens, R., Look, M. P., Van Deurzen, C. H. M., Span, P. N., Sweep, F. C. G. J. et al. (2014). Ferritin heavy chain in triple negative breast cancer: A favorable prognostic marker that relates to a cluster of differentiation 8 positive (CD8+) effector T-cell response. *Mol. Cell. Proteomics* 13, 1814-1827. 10.1074/mcp.M113.037176 24742827 PMC4083117

[DMM049286C44] Lyons, T. R., O'Brien, J., Borges, V. F., Conklin, M. W., Keely, P. J., Eliceiri, K. W., Marusyk, A., Tan, A. C. and Schedin, P. (2011). Postpartum mammary gland involution drives progression of ductal carcinoma in situ through collagen and COX-2. *Nat. Med.* 17, 1109-1116. 10.1038/nm.2416 21822285 PMC3888478

[DMM049286C45] Martinson, H. A., Jindal, S., Durand-Rougely, C., Borges, V. F. and Schedin, P. (2015). Wound healing-like immune program facilitates postpartum mammary gland involution and tumor progression. *Int. J. Cancer* 136, 1803-1813. 10.1002/ijc.29181 25187059 PMC4324053

[DMM049286C46] McDaniel, S. M., Rumer, K. K., Biroc, S. L., Metz, R. P., Singh, M., Porter, W. and Schedin, P. (2006). Remodeling of the mammary microenvironment after lactation promotes breast tumor cell metastasis. *Am. J. Pathol.* 168, 608-620. 10.2353/ajpath.2006.050677 16436674 PMC1606507

[DMM049286C47] Mulligan, L. M. (2019). GDNF and the RET receptor in cancer: new insights and therapeutic potential. *Front. Physiol.* 10, 1873. 10.3389/fphys.2018.01873 PMC6330338 30666215

[DMM049286C48] Nguyen, A. V. and Pollard, J. W. (2000). Transforming growth factor β3 induces cell death during the first stage of mammary gland involution. *Development* 127, 3107-3118. 10.1242/dev.127.14.3107 10862748

[DMM049286C49] O'Brien, J., Lyons, T., Monks, J., Lucia, M. S., Wilson, R. S., Hines, L., Man, Y.-G., Borges, V. and Schedin, P. (2010). Alternatively activated macrophages and collagen remodeling characterize the postpartum involuting mammary gland across species. *Am. J. Pathol.* 176, 1241-1255. 10.2353/ajpath.2010.090735 20110414 PMC2832146

[DMM049286C50] Palmer, J. R., Viscidi, E., Troester, M. A., Hong, C.-C., Schedin, P., Bethea, T. N., Bandera, E. V., Borges, V., McKinnon, C., Haiman, C. A. et al. (2014). Parity, lactation, and breast cancer subtypes in African American Women: Results from the AMBER Consortium. *J. Natl. Cancer Inst.* 106, dju237. 10.1093/jnci/dju237 25224496 PMC4271113

[DMM049286C51] Paratala, B. S., Chung, J. H., Williams, C. B., Yilmazel, B., Petrosky, W., Williams, K., Schrock, A. B., Gay, L. M., Lee, E., Dolfi, S. C. et al. (2018). RET rearrangements are actionable alterations in breast cancer. *Nat. Commun.* 9, 4821. 10.1038/s41467-018-07341-4 30446652 PMC6240119

[DMM049286C52] Pennock, N. D., Martinson, H. A., Guo, Q., Betts, C. B., Jindal, S., Tsujikawa, T., Coussens, L. M., Borges, V. F. and Schedin, P. (2018). Ibuprofen supports macrophage differentiation, T cell recruitment, and tumor suppression in a model of postpartum breast cancer. *J. Immunother. Cancer* 6, 98. 10.1186/s40425-018-0406-y 30285905 PMC6167844

[DMM049286C53] Pensa, S., Watson, C. J. and Poli, V. (2009). Stat3 and the inflammation/acute phase response in involution and breast cancer. *J. Mammary Gland Biol. Neoplasia* 14, 121-129. 10.1007/s10911-009-9124-x 19424782

[DMM049286C54] Perea, D., Guiu, J., Hudry, B., Konstantinidou, C., Milona, A., Hadjieconomou, D., Carroll, T., Hoyer, N., Natarajan, D., Kallijärvi, J. et al. (2017). Ret receptor tyrosine kinase sustains proliferation and tissue maturation in intestinal epithelia. *EMBO J.* 36, 3029-3045. 10.15252/embj.201696247 28899900 PMC5641678

[DMM049286C55] Plaza-Menacho, I., Morandi, A., Mologni, L., Boender, P., Gambacorti-Passerini, C., Magee, A. I., Hofstra, R. M. W., Knowles, P., McDonald, N. Q. and Isacke, C. M. (2011). Focal Adhesion Kinase (FAK) binds RET kinase via its FERM domain, priming a direct and reciprocal RET-FAK transactivation mechanism. *J. Biol. Chem.* 286, 17292-17302. 10.1074/jbc.M110.168500 21454698 PMC3089571

[DMM049286C56] Quaglino, A., Salierno, M., Pellegrotti, J., Rubinstein, N. and Kordon, E. C. (2009). Mechanical strain induces involution-associated events in mammary epithelial cells. *BMC Cell Biol.* 10, 55. 10.1186/1471-2121-10-55 19615079 PMC2721828

[DMM049286C57] Radisky, D. C. and Hartmann, L. C. (2009). Mammary involution and breast cancer risk: Transgenic models and clinical studies. *J. Mammary Gland Biol. Neoplasia* 14, 181-191. 10.1007/s10911-009-9123-y 19404726 PMC2693781

[DMM049286C58] Rädler, P. D., Wehde, B. L. and Wagner, K. U. (2017). Crosstalk between STAT5 activation and PI3K/AKT functions in normal and transformed mammary epithelial cells. *Mol. Cell. Endocrinol.* 451, 31-39. 10.1016/j.mce.2017.04.025 28495456 PMC5515553

[DMM049286C78] Ramirez-Ardila, D. E., Ruigrok-Ritstier, K., Helmijr, J. C., Look, M. P., van Laere, S., Dirix, L., Berns, E. M. J. J. and Jansen, M. P. H. M. (2016). LRG1 mRNA expression in breast cancer associates with PIK3CA genotype and with aromatase inhibitor therapy outcome. *Mol. Oncol*. 10, 1363-1373. 10.1016/j.molonc.2016.07.004 27491861 PMC5423197

[DMM049286C59] Schedin, P. (2006). Pregnancy-associated breast cancer and metastasis. *Nat. Rev. Cancer* 6, 281-291. 10.1038/nrc1839 16557280

[DMM049286C60] Schedin, P., O'Brien, J., Rudolph, M., Stein, T. and Borges, V. (2007). Microenvironment of the involuting mammary gland mediates mammary cancer progression. *J. Mammary Gland Biol. Neoplasia* 12, 71-82. 10.1007/s10911-007-9039-3 17318269

[DMM049286C61] Schere-Levy, C., Buggiano, V., Quaglino, A., Gattelli, A., Cirio, M. C., Piazzon, I., Vanzulli, S. and Kordon, E. C. (2003). Leukemia inhibitory factor induces apoptosis of the mammary epithelial cells and participates in mouse mammary gland involution. *Exp. Cell Res.* 282, 35-47. 10.1006/excr.2002.5666 12490192

[DMM049286C62] Spanheimer, P. M., Park, J. M., Askeland, R. W., Kulak, M. V., Woodfield, G. W., De Andrade, J. P., Cyr, A. R., Sugg, S. L., Thomas, A. and Weigel, R. J. (2014). Inhibition of RET increases the efficacy of antiestrogen and is a novel treatment strategy for luminal breast cancer. *Clin. Cancer Res.* 20, 2115-2125. 10.1158/1078-0432.CCR-13-2221 24526731 PMC3989441

[DMM049286C63] Stein, T., Morris, J. S., Davies, C. R., Weber-Hall, S. J., Duffy, M.-A., Heath, V. J., Bell, A. K., Ferrier, R. K., Sandilands, G. P. and Gusterson, B. A. (2004). Involution of the mouse mammary gland is associated with an immune cascade and an acute-phase response, involving LBP, CD14 and STAT3. *Breast Cancer Res.* 6, R75-R91. 10.1186/bcr753 14979920 PMC400652

[DMM049286C64] Stein, T., Salomonis, N. and Gusterson, B. A. (2007). Mammary gland involution as a multi-step process. *J. Mammary Gland Biol. Neoplasia* 12, 25-35. 10.1007/s10911-007-9035-7 17431797

[DMM049286C65] Stewart, T. A., Pattengale, P. K. and Leder, P. (1984). Spontaneous mammary adenocarcinomas in transgenic mice that carry and express MTV/myc fusion genes. *Cell* 38, 627-637. 10.1016/0092-8674(84)90257-5 6488314

[DMM049286C66] Varešlija, D., Priedigkeit, N., Fagan, A., Purcell, S., Cosgrove, N., O'Halloran, P. J., Ward, E., Cocchiglia, S., Hartmaier, R., Castro, C. A. et al. (2019). Transcriptome characterization of matched primary breast and brain metastatic tumors to detect novel actionable targets. *J. Natl. Cancer Inst.* 111, 388-398. 10.1093/jnci/djy110 29961873 PMC6449168

[DMM049286C67] Watson, C. J. (2001). Stat transcription factors in mammary gland development and tumorigenesis. *J. Mammary Gland Biol. Neoplasia* 6, 115-127. 10.1023/A:1009524817155 11467447

[DMM049286C68] Watson, C. J. (2006). Post-lactational mammary gland regression: molecular basis and implications for breast cancer. *Expert Rev. Mol. Med.* 8, 1-15. 10.1017/S1462399406000196 17178008

[DMM049286C69] Watson, C. J. and Khaled, W. T. (2008). Mammary development in the embryo and adult: a journey of morphogenesis and commitment. *Development* 135, 995-1003. 10.1242/dev.005439 18296651

[DMM049286C70] Williams, M. M. and Cook, R. S. (2015). Bcl-2 family proteins in breast development and cancer: Could Mcl-1 targeting overcome therapeutic resistance? *Oncotarget* 6, 3519-3530. 10.18632/oncotarget.2792 25784482 PMC4414133

[DMM049286C71] Williams, C., Helguero, L., Edvardsson, K., Haldosén, L. A. and Gustafsson, J. A. (2009). Gene expression in murine mammary epithelial stem cell-like cells shows similarities to human breast cancer gene expression. *Breast Cancer Res.* 11, R26. 10.1186/bcr2256 19426500 PMC2716494

