# Supplementary material for: Aberrant RET expression affects normal mammary gland post-lactation transition, enhancing cancer potential
Source: Dis Model Mech. 2022 Mar 29;15(3):dmm049286. doi: 10.1242/dmm.049286 (PMC8990024; doi:10.1242/dmm.049286)
Supplement: Supplementary information [file dmm-15-049286-s1.pdf]

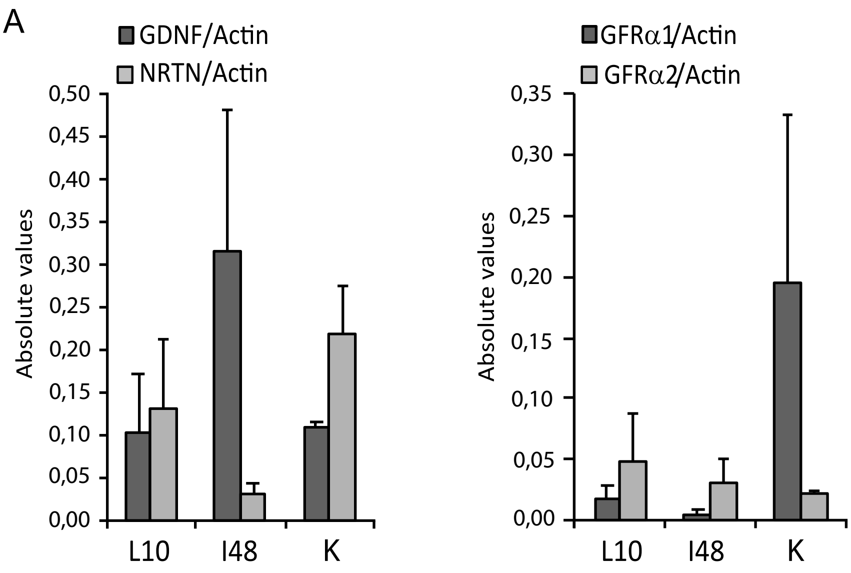

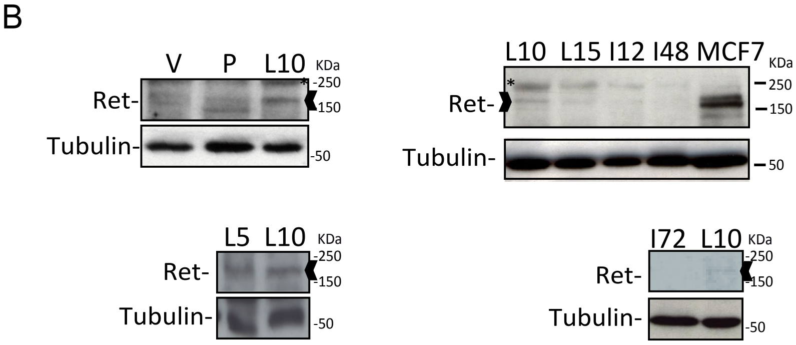

Fig. S1. RET signalling partner expression during mammary gland development. A. Levels of mRNA expression for RET ligands *GDNF* and neurturin (*NRTN*) and the co-receptors *GFRα1* and *GFRα2* (n=2-3) normalized with actin as a housekeeping gene were analyzed by qRT-PCR in mammary tissue from female mice at lactating (L10) and involution (I48). A sample from mouse kidney (K) was used as a positive control. Corresponding quantification is shown as the mean ± s.e. **B.** For each mammary gland developmental stage a representative band from WB assays detecting RET expression is shown. As a positive control, a lysate from 10 days lactating mammary tissue (L10) is showed in each blot. A lysate from MCF7 breast cancer cells is included in a WB as a control for human RET expression, in dicating that in mouse tissue one of the human RET bands is absent. For mouse tissue an unidentified band is indicated by asterisk (*). Small arrows on the blots indicate the specifi c band used in the corresponding Ret quantification.

Disease Models & Mechanisms • Supplementary information

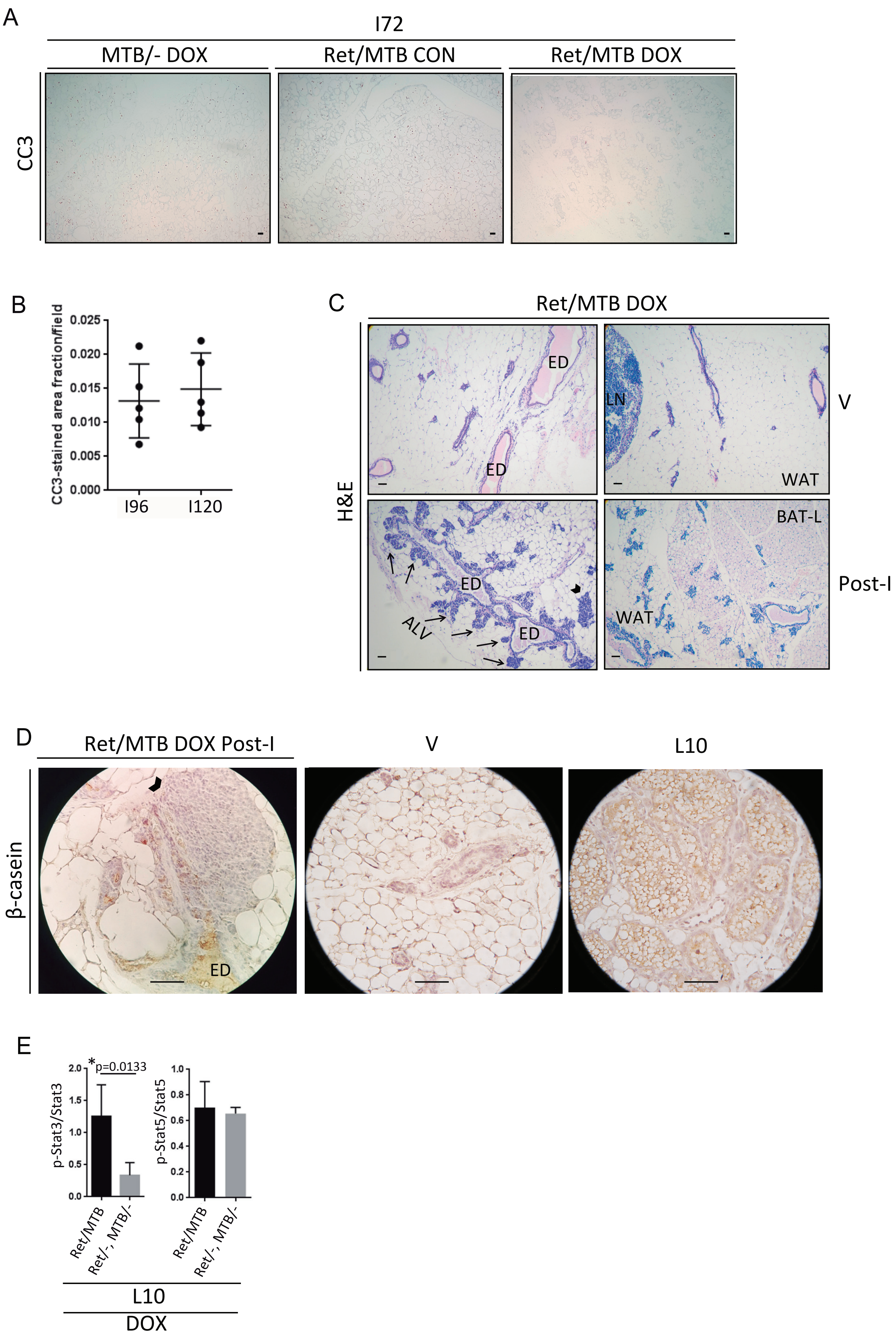
A
I72
MTB/- DOX
Ret/MTB CON
Ret/MTB DOX
CC3
B
CC3-stained area fraction/field
0.025
0.020
0.015
0.010
0.005
0.000
I96
I120
C
Ret/MTB DOX
H&E
ED
ED
LN
WAT
V
ALV
ED
ED
BAT-L
WAT
Post-I
D
Ret/MTB DOX Post-I
V
L10
β-casein
ED
E
*p=0.0133
p-Stat3/Stat3
2.0
1.5
1.0
0.5
0.0
p-Stat5/Stat5
1.0
0.8
0.6
0.4
0.2
0.0
Ret/MTB
Ret/-, MTB/-
Ret/MTB
Ret/-, MTB/-
L10
DOX

Fig. S2. Remodelling alterations induced by RET overexpression. A. A lower magnification of CC3-stained sections from specimens depicted in **Fig. 3** are shown. Scale bars=50µm**. B.** Quantification of IHC for specific CC3-stained sections of none transgenic mammary glands at different times of involution, I96 (n=5) and I120 (n=5) (involution 96 and 120 hours, respectively), is shown. Data presented are the mean ± s.d. **C**. Morphology features by H&E-staining. Scale bars=50µm. **D.** IHC analysis for β-casein expression in mammary gland tissue taken from animals used in **Fig. 4** in the indicated conditions. Neoplastic lesions, mammary intra-epithelial neoplasia, are indicated by arrowheads. WAT: white adipose tissue; BAT-L: brown-like adipose tissue; ED: ectatic duct; ALV: persistent alveoli. Scale bars=50µm. **E**. Quantification of the WB depicted in **Fig. 5C** showing the analyses for Stat3 and Stat5 activation in DOX-treated RET-expressing Ret/MTB 10 days lactating (L10) gland lysates (n=4) and the corresponding controls (Ret/-, MTB/-) (n=4) is shown. For WB quantification analysis was performed using imageJ for the indicated protein/protein ratios. Data shown are the mean ± s.e.m. p value by unpaired t test.

Disease Models & Mechanisms • Supplementary information

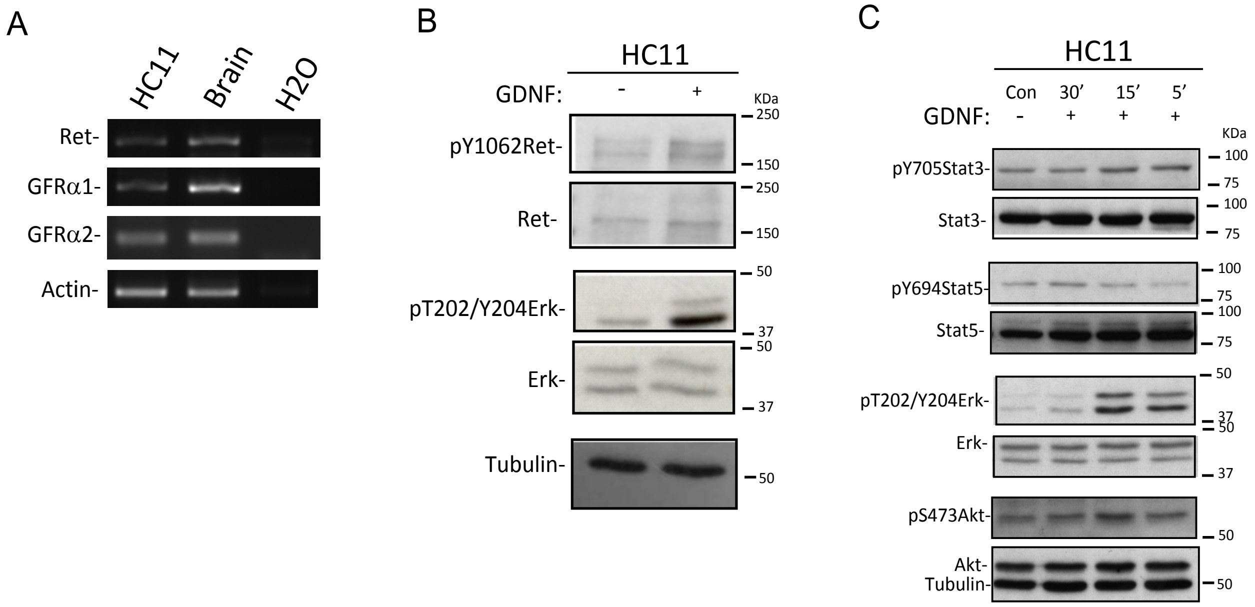

Fig. S3. RET-driven signalling in Ret-expressing mammary epithelial cells. A. *Ret*, *GFRα1* and *GFRα2* mRNA expression is shown for HC11 cells by RT-PCR. Detection of *Actin* mRNA was used as a housekeeping gene. **B.** A WB assay shows the pY1062Ret/Ret protein levels in HC11 cells after 5 minutes of GDNF stimulation (10ng/ml). Phosphorylation levels of Erk (pT202Y204Erk/Erk) are shown as positive control of activation of RET downstream signalling. Tubulin is used as a loading control. **C.** WBs show the phospho/total signaling protein levels for the indicated antibodies for lysates from GDNF-treated (10ng/ml) (+) HC11 cultures at different times of stimulation (5, 15 and 30 minutes) compared with untreated cells (-).

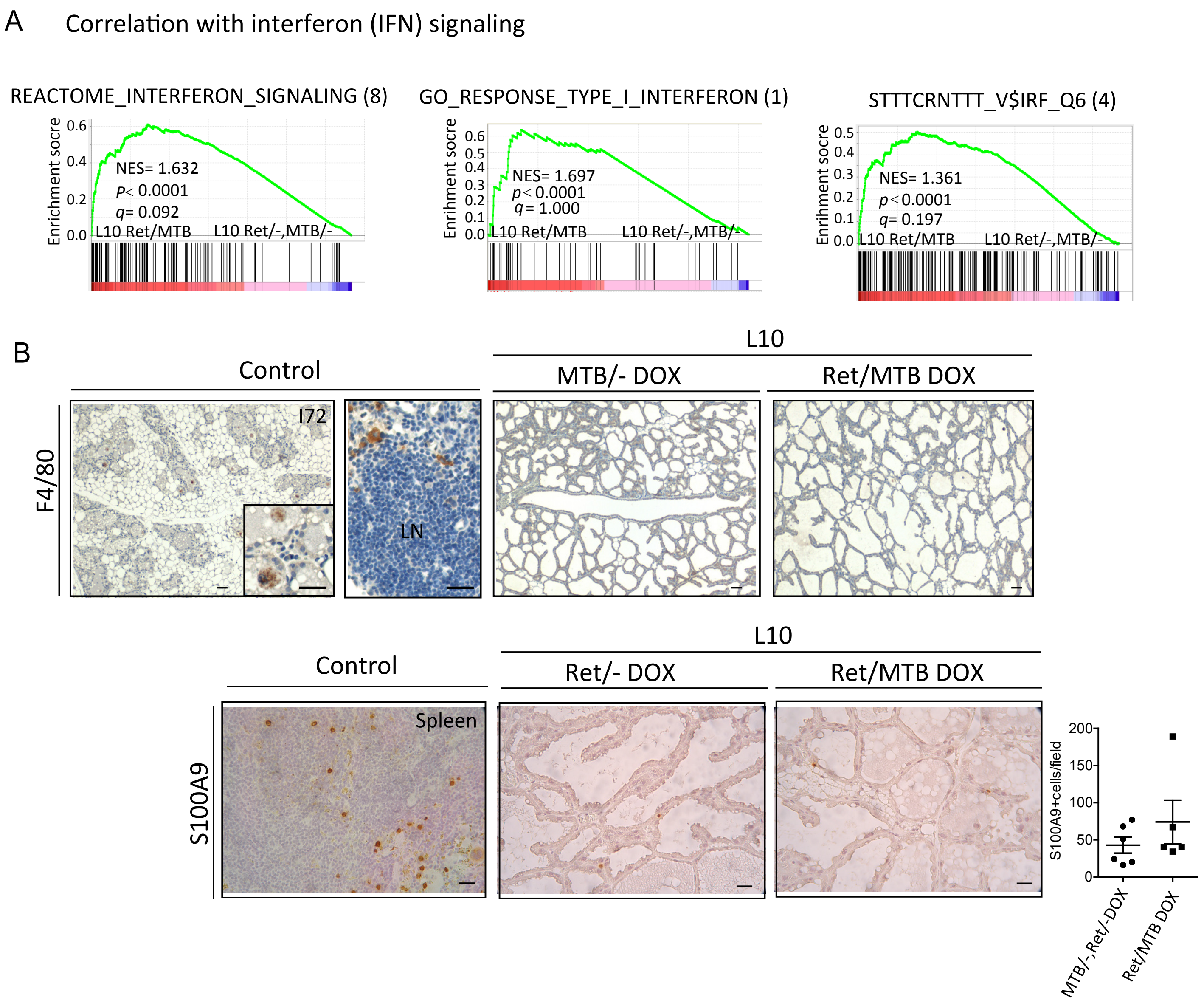

Fig. S4. Additional analysis pertaining to Fig. 6. A. GSEA shows the correlation between indicators of an interferon signaling signatures along canonical pathways, biological process and transcription factors (REACTOME_INTERFERON_SIGNALING, GO_RESPONSE_TO_TYPE_I_INTERFERON and STTTCRNTTT_V$IRF_Q6, respectively) and gene sets up- or downregulated in the Ret/MTB (n=6) or control (n=6) (Ret/-, MTB/-) 10 days lactating glands. NES: normalized enrichment score; q: false discovery rate. p-value was computed as implemented in GSEA. **B.** Mammary gland sections from Ret/- or MTB/- and Ret/MTB females at 10 days of lactation (L10) following treatment with doxycycline (2g/l) for 8 days were analyzed by IHC for either F4/80 or S100A9. For S100A9 data are presented as the mean ± s.d. and quantification is shown (n=6 each group). Sections from either spleen or lymph node (LN) were used as positive control for the corresponding antibodies. Scale bars=50µm.

Disease Models & Mechanisms • Supplementary information

Table S1. List of genes regulated by Ret overexpression in lactation.

Symbol	Gene	[a]2LogFC	GO biological process
Ace	Angiotensin I converting enzyme 1	3,11	Angiotensin maturation
Adhfe1	Alcohol dehydrogenase iron containing 1	1,42	Glutamate degradation via 2-oxoglutarate
Antxrl	Anthrax toxin receptor-like	1,92	Toxin transport
Arhgef12	Rho guanine nucleotide exchange factor 12	0,84	Rho protein signal transduction
Basp1	Brain abundant membrane attached signal protein 1	2,58	Downregulation of transcription
Bcl2l1	BCL2-like 1	3,68	Apoptotic mitochondrial changes
Btla	B and T lymphocyte associated	3,15	Adaptive immune response
B2m	Beta-2 microglobulin	2,55	Immune response
Capn12	Calpain 12	0,59	Proteolysis
Ccdc167	Coiled-coil domain-containing 167	0,55	---
Cep85	Centrosomal protein 85	1,67	Chromosome segregation
Chd4	Chromodomain helicase DNA binding protein 4	2,42	Chromatin organization
Clca3a1	Chloride channel accessory 3A1	3,54	Chloride transport
Clca3a2	Chloride channel accessory 3A2	3,28	Extrinsic apoptosis in absence of ligand
Cnbd2	Cyclic nucleotide binding domain containing 2	2,5	Spermatogenesis
Cttnbp2nl	CTTNBP2 N-terminal-like	2,39	Down-regulation of transmembrane transport
C030034L19Rik	RIKEN cDNA C030034L19 gene	1,18	---
C4b	Complement component 4B	2,64	Complement activation
Dst	Dystonin	2,88	Cell motility and adhesion
Dtx1	Deltex 1-E3 ubiquitin ligase	2,04	Cell surface receptor signaling pathway
Dvl1	Dishevelled segment polarity protein 1	1,08	Axon extension / Wnt signaling pathway
Ermard	ER membrane-associated RNA degradation	0,7	Multicellular organism development
Fbl	Fibrillarin	1,1	rRNA methylation
Fis1	Fission mitochondrial 1	2,37	Apoptotic process
Fth1	Ferritin heavy polypeptide 1	1,69	Ferric ion transport
Gls	Glutaminase	2,78	Glutamate homeostasis / Suckling behavior
Gm11651	Predicted gene 11651	2,77	---
Gm16907	Predicted gene 16907	0,88	---

Disease Models & Mechanisms • Supplementary information

Gm20319	Predicted gene 20319	0,85	---
Gm3435	Predicted gene 3435	0,77	---
Hcfc1r1	Host cell factor C1 regulator 1	1,25	---
Hnrnpa3	Heterogeneous nuclear ribonucleoprotein A3	2,67	mRNA processing
Hps3	HPS3, biogenesis of lysosomal organelles complex 2 subunit 1	1,04	Organelle organization
Hrct1	Histidine rich carboxyl terminus 1	2,71	---
Ift172	Intraflagellar transport 172	1,11	Determination of left/right symmetry
Lcn2	Lipocalin 2	2,75	Extrinsic apoptotic signaling pathway
Lifr	Leukemia inhibitory factor receptor	1,64	Response to cytokine
Lipe	Lipase hormone-sensitive	2,24	Lipid catabolic process
Lipt1	Lipoyltransferase 1	1,27	Protein lipoylation
Lrg1	Leucine-rich alpha-2-glycoprotein 1	1,93	Brown fat cell differentiation / Up-regulation of angiogenesis
Ltf	Lactotransferrin	1,99	Innate immune response in mucosa
Mfge8	Milk fat globule-EGF factor 8 protein	0,97	Angiogenesis
Nop9	NOP9 Nucleolar Protein	2,85	---
Nrbp1	Nuclear receptor binding protein 1	1,33	ER to Golgi vesicle-mediated transport
Oas1h	2'-5' oligoadenylate synthetase 1H	3,82	Immune response
Ogfr	Opioid growth factor receptor	1,2	Regulation of growth
Parp14	Poly (ADP-ribose) polymerase family member 14	2,77	Negative regulation of gene expression
Pdap1	PDGFA associated protein 1	0,9	Signal transduction
Pigh	Phosphatidylinositol glycan anchor biosynthesis class H	2,23	Cellular protein modification process
Pip	Prolactin induced protein	3,54	Regulation of immune system process
Pkm	Pyruvate kinase muscle	1,25	ATP biosynthetic process
Plet1	Placenta expressed transcript 1	1,17	Cell differentiation
Plet1os	Placenta expressed transcript 1 (opposite strand)	0,99	---
Ppt1	Palmitoyl-protein thioesterase 1	0,59	Lipid catabolic process
Prickle3	Prickle planar cell polarity protein 3	0,68	Cell projection organization
Prune2	Prune homolog 2	2,04	Apoptotic process
Ptgdr2	Prostaglandin D2 receptor 2	1,36	Calcium-mediated signaling
Rell2	RELT-like 2	1,75	Positive regulation of cell-substrate adhesion
Saa1	Serum amyloid A 1	5,23	Acute inflammatory response

Disease Models & Mechanisms • Supplementary information

Saa2	Serum amyloid A 2	6,46	Acute inflammatory response
Serinc3	Serine incorporator 3	1,18	Sphingolipid metabolic process
Setdb1	SET domain, bifurcated 1	1,34	Histone H3-K9 methylation
Slc6a4	Solute carrier family 6 member 4	2,85	Neurotransmitter transport
Sorbs2os	Sorbin and SH3 domain containing 2 (opposite strand)	2,56	---
Sufu	SUFU negative regulator of Hedgehog signaling	3,85	Negative regulation of transcription (RNA pol II)
Syt12	Synaptotagmin XII	1,25	Calcium-ion regulated exocytosis
Tagln	Transgelin	0,74	Epithelial cell differentiation
Tanc2	Tetratricopeptide repeat, ankyrin repeat and coiled-coil containing 2	1,77	In utero embryonic development
Tmem189	Transmembrane protein 189	0,75	Lipid metabolic process
Tor1aip2	Torsin A interacting protein 2	0,67	Endoplasmic reticulum organization
Trim12c	Tripartite motif-containing 12C	0,82	Autophagy / Innate immune response
Trim14	Tripartite motif-containing 14	1,66	Innate immune response
Trim34a	Tripartite motif-containing 34A	1,13	Autophagy / Innate immune response
Trpv6	Transient receptor potential cation channel subfamily V member 6	1,64	Calcium ion transport
Ttn	Titin	2,69	Heart development
Zdhhc6	Zinc finger DHHC domain-containing 6	0,53	Protein targeting to membrane
Zfp513	Zinc finger protein 513	2,88	Regulation of transcription
Zmynd15	Zinc finger MYND-type containing 15	0,91	Transcription repressor activity
Znrf1	Zinc and ring finger 1	0,45	Cytoskeleton organization
Zpbp2	Zona pellucida binding protein 2	3,52	Binding of sperm to zona pellucida
1700088E04Rik	RIKEN cDNA 1700088E04 gene	0,92	---
1810062O18Rik	RIKEN cDNA 1810062O18 gene	1,82	---
2410017I17Rik	RIKEN cDNA 2410017I17 gene	0,72	---
3110035E14Rik	RIKEN cDNA 3110035E14 gene	1,83	---
4930455H04Rik	RIKEN cDNA 4930455H04 gene	2,33	---
6030443J06Rik	RIKEN cDNA 6030443J06 gene	0,74	---
9030025P20Rik	RIKEN cDNA 9030025P20 gene	0,92	---
Acp2	Acid phosphatase 2 lysosomal	-0,49	Autophagic cell death
Adamts5	A disintegrin-like and metallopeptidase with thrombospondin type 1 motif, 5	-1,99	Extracellular matrix disassembly
Aida	Axin interactor dorsalization associated	-1,47	Dorsal/ventral pattern formation
Akap2	A kinase anchor protein 2	-3,13	---

Arvcf	Armadillo repeat gene deleted in velocardiofacial syndrome	-0,82	Cell-cell adhesion
Atg9b	Autophagy related 9B	-1,44	Autophagosome assembly
AW146154	Expressed sequence AW146154	-1,54	---
Bri3	Brain protein I3	-1,2	Neutrophil degranulation
B3gnt8	UDP-GlcNAc:betaGal beta-1,3-N-acetylglucosaminyltransferase 8	-0,43	Protein glycosylation
Capn7	Calpain 7	-1,01	Proteolysis
Ccdc73	Coiled-coil domain containing 73	-0,71	---
Cdh23	Cadherin 23	-1,96	Locomotory behavior
Cmtr1	Cap methyltransferase 1	-0,49	7-methylguanosine mRNA capping
Ctnna1	Catenin alpha 1	-2,89	Epithelial cell-cell adhesion
Ctu2	Cytosolic thiouridylase subunit 2	-0,91	tRNA thio-modification
Dcps	Decapping enzyme scavenger	-1,04	mRNA processing
DXBay18	DNA segment, Chr X, Baylor 18	-2,23	---
Gm13710	Predicted gene 13710	-0,93	---
Gm15441	Predicted gene 15441	-0,52	---
Gnal	Guanine nucleotide binding protein, alpha stimulating, olfactory type	-0,84	G protein-coupled receptor signaling pathway
Gpr62	G protein-coupled receptor 62	-1,66	G protein-coupled receptor signaling pathway
Gtf3c5	General transcription factor IIIC, polypeptide 5	-1,80	Transcription initiation from Pol III promoter
Hbp1	High mobility group box transcription factor 1	-0,53	Negative regulation of lipid transport
Hoxb3	Homeobox B3	-0,99	Angiogenesis
H2-T10	Histocompatibility 2, T region locus 10	-0,51	Positive regulation of T cell mediated cytotoxicity
Iars2	Isoleucine-tRNA synthetase 2 mitochondrial	-2,24	Isoleucyl-tRNA aminoacylation
Icmt	Isoprenylcysteine carboxyl methyltransferase	-1,63	Protein tagging activity
Jmjd8	Jumonji domain containing 8	-0,27	Regulation of glycolytic process
Lamb2	Laminin beta 2	-2,97	Cell migration
Lrrc61	Leucine rich repeat containing 61	-0,76	Protein localization
Ltbp2	Latent transforming growth factor beta binding protein 2	-1,48	Supramolecular fiber organization
Mapk8ip3	Mitogen-activated protein kinase 8 interacting protein 3	-0,36	Activation of JUN kinase activity

Disease Models & Mechanisms • Supplementary information

Mat2a	Methionine adenosyltransferase II alpha	-0,79	Cellular response to leukemia inhibitory factor
Nat8f3	N-acetyltransferase 8 family member 3	-3,08	Histone acetylation
Notch4	Notch 4	-1,33	Mammary gland development
Pakap	Paralemmin A kinase anchor protein	-3,13	Regulation of cell shape
Prrc2b	Proline-rich coiled-coil 2B	-1,59	Cell differentiation
Rai1	Retinoic acid induced 1	-1,22	Regulation of transcription by RNA polymerase II
Rasgrp4	RAS guanyl releasing protein 4	-0,47	Cell differentiation
Rgp1	RAB6A GEF complex partner 1	-2,12	Negative regulation of cyclin catabolism
Rnf146	Ring finger protein 146	-1,22	Wnt signaling pathway
Rrp7a	Ribosomal RNA processing 7 homolog A	-0,48	rRNA processing
Selp	Selectin, platelet	-3,5	Cell adhesion / Inflammatory response
Sgsh	N-sulfoglucosamine sulfohydrolase	-1,54	Glycosaminoglycan degradation
Slc5a2	Solute carrier family 5 member 2	-0,44	Glucose transmembrane transport / Sodium ion transport
Slco2b1	Solute carrier organic anion transporter family member 2b1	-0,9	Organic anion transport
Smpd4	Sphingomyelin phosphodiesterase 4	-1,09	Cellular response to tumor necrosis factor
Sphk2	Sphingosine kinase 2	-1,05	Sphingosine biosynthetic process
Spout1	SPOUT domain containing methyltransferase 1	-0,78	Production of miRNAs involved in gene silencing by miRNA
Syn3	Synapsin III	-0,37	Neurotransmitter secretion
Tmem102	Transmembrane protein 102	-2,47	Positive regulation of T cell migration
Tmem88	Transmembrane protein 88	-0,68	Wnt signaling pathway
Tnfsf13	Tumor necrosis factor ligand superfamily member 13	-0,98	Regulation of immune response
Toposr	Topoisomerase I binding arginine/serine-rich	-0,84	Protein polyubiquitination
Tubgcp6	Tubulin gamma complex associated protein 6	-0,47	Microtubule nucleation
Wdr38	WD repeat domain 38	-0,78	Hematopoietic progenitor cell differentiation
Zbtb40	Zinc finger and BTB domain containing 40	-1,39	Cellular response to DNA damage stimulus
Zfp971	Zinc finger protein 971	-2,32	---

Disease Models & Mechanisms • Supplementary information

2610203C22Rik	RIKEN cDNA 2610203C22 gene	-1,35	---
4930592A05Rik	RIKEN cDNA 4930592A05 gene	-0,6	---
9430069I07Rik	RIKEN cDNA 9430069I07 gene	-3,19	---

[a]2LogFC: log 2 of fold change; a value of 1,5 for 2LogFC means 3-fold change. ---: GO not reported

Disease Models & Mechanisms • Supplementary information
